# Oxygen Reduction Reaction on N‑Doped Graphene under Acidic Conditions: Integrating N‑Dopant Site Structure Changes with Competing Elementary Reaction Steps

**DOI:** 10.1021/acs.jpcc.6c04248

**Published:** 2026-09-23

**Authors:** Spand Mehta, Aishwarya Rao, Aravind Asthagiri

**Affiliations:** William G. Lowrie Department of Chemical and Biomolecular Engineering, 2647 The Ohio State University, 151 W. Woodruff Avenue, Columbus, Ohio 43210, United States

## Abstract

Nitrogen-doped carbon (CN_*x*_) catalysts are promising alternatives to replace Pt for the electrochemical oxygen reduction reaction (ORR) in proton-exchange-membrane fuel cells. To date, there remains an ongoing debate regarding the nature of active sites on CN_*x*_. Experimental half-cell ORR activity studies of CN_*x*_ catalysts correlate the fraction of pyridinic sites (characterized ex situ by X-ray photoelectron spectroscopy) to ORR activity. In contrast, density functional theory (DFT) studies using the chemical hydrogen electrode (CHE) approach predominantly show quaternary/oxide sites to have higher activity than that of pyridinic sites. In this work, we integrate the initial thermodynamic stability analysis of the N-dopant structure at 1.23 V-RHE with subsequent competing ORR elementary steps as a function of applied potential. In contrast to earlier studies, our integrated approach predicts high activity for pyridinic sites, with a majority of quaternary sites, except basal quaternary, which show low activity. This enhanced activity occurs through an initial oxidation of the pyridinic sites via H_2_O that converts the pyridinic sites to pyridinic oxide structures at 1.23 V-RHE (before the start of ORR). Subsequent ORR steps on this structure result in a stable 4 proton–electron cycle of elementary steps that yield high ORR DFT thermodynamic limiting potentials. Therefore, the DFT-CHE results suggest that pyridinic sites are indeed active for ORR but first must convert to an oxidized structure that mediates the ORR mechanism. Our findings suggest a potential mechanism to reconcile the ongoing debate on the nature of active sites and provide fundamental insights into the ORR activity of CN_*x*_ catalysts.

## Graphical abstract

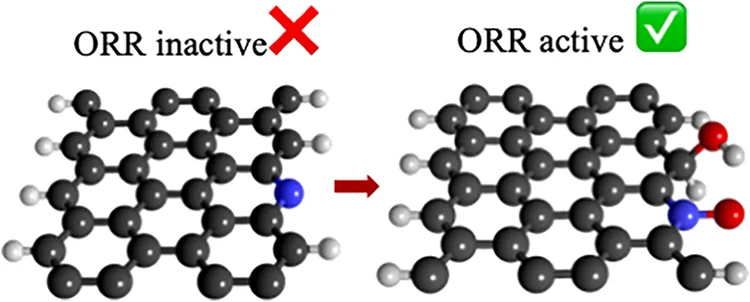

## Introduction

Proton-exchange membrane fuel cells (PEMFCs) are considered to be an attractive low-carbon energy conversion technology to overcome an ever-increasing energy crisis and environmental pollution.^1^ The oxygen reduction reaction (ORR) has been studied extensively due to its application as a cathodic reaction in PEMFCs.^2^ However, ORR suffers from sluggish kinetics, and the development of cost-effective ORR catalysts with high electrochemical performance remains a challenge. Despite the excellent electrochemical activity of the state-of-the-art Pt catalysts, they have high costs, limited abundance, and susceptibility to CO poisoning. Further, Pt is prone to leaching under the acidic operating conditions of PEMFCs.^3,4^ Nonprecious metal catalysts (NPMCs) and metal-free catalysts have emerged as alternative candidates for ORR catalysts.^5,6^ In recent years, there has been active research on metal–nitrogen-carbon (MeNC) catalysts with transition metal (Me) coordinated with N as active sites.^7–9^ Heteroatom-doped (S, P, N, B) carbon materials also show remarkable ORR activities.^10–15^ In particular, nitrogen-doped carbon (CN_*x*_) catalysts have been extensively studied and show excellent electrochemical performance, conductivity, mechanical properties, and electrochemical stability to leaching under acidic conditions.^14,16^ CN_*x*_ catalysts also have been shown to be resistant to several poisons (CO, CN^–^, Cl^–^, and H_2_S) that readily poison Pt-based and FeCN catalysts.^17–22^ These poisoning studies also established that CN_*x*_ catalysts are distinct from MeNC and any metal used in the synthesis of CN_*x*_ is removed or is inaccessible.

However, significant improvement in CN_*x*_ catalysts is required to achieve the necessary electrocatalytic performance to compete with platinum and achieve commercial viability. Such an improvement will require a fundamental understanding of the active sites in CN_*x*_ catalysts. Because the CN_*x*_ material is heterogeneous with a range of local coordination for the N-dopant, the primary characterization tool has been X-ray photoelectron spectroscopy (XPS), focused on the N 1s spectra, often fitted to three main peaks corresponding to quaternary, pyridinic, and pyridinic oxide nitrogen. Focusing on acidic conditions, Ozkan and co-workers have reported that the pyridinic site is correlated with the ORR. In earlier studies, they showed changing the growth catalyst and N precursor results in different CN_*x*_ structures that can vary from exposing more basal planes (onion or stacked cups) versus edge structures (stacked platelets) with the latter showing higher ORR activity.^23^ In a later study, they identified a synthesis procedure (nitrogen precursor/growth catalyst) where changing just the pyrolysis temperature could induce CN_*x*_ catalysts with varying ratios of pyridinic, quaternary, and pyridinic oxide while keeping the dopant concentration of N in the CN_*x*_ catalyst constant. These CN_*x*_ catalysts showed a linear correlation between ORR activity and relative pyridinic N content.^14^ Nakamura and co-workers used highly oriented pyrolytic graphite that was varied in pyridinic vs quaternary N and found ORR activity was correlated to the pyridinic N sites.^24,25^

Density functional theory (DFT) modeling of the graphene-doped catalyst involves consideration of different nitrogen-doped sites^26–30^ and codoping of other atoms.^10,11,31^ Previous studies have calculated the DFT activity of different possible active sites by considering the associative and dissociative ORR mechanisms.^26,32,11,33–37^ The DFT studies of CN_*x*_ predict carbon adjacent to the nitrogen atom to be the active site for ORR, with quaternary models showing the highest thermodynamic limiting potentials. However, this contrasts with experimental results that have shown pyridinic sites to be a marker for ORR activity.^14,23–25,38^

A key feature missing from these DFT studies is the potential changes to the CN_*x*_ site structure due to surface protonation/oxidation of N species. Instead, the earlier studies treat the bare, protonated, and N-oxide species as independent site models.^11,36,39,40^ The importance of incorporating surface stability has been shown in DFT ORR studies on metal surfaces, where surface Pourbaix diagrams are generated to examine the stability of oxidized/protonated structures as a function of applied potential and pH.^41,42^ In the current paper, we first evaluate the surface stability of different pyridinic/quaternary sites of CN_*x*_ in the absence of molecular oxygen (at a pH of zero for acidic conditions). Using the DFT-predicted thermodynamic surface at 1.23 V-RHE, we then sequentially evaluated the ORR elementary steps to convert O_2_ to H_2_O on the surface. However, in this process, for each step, we consider alternative pathways, including those that allow for additional O_2_ to be reduced on the surface. Such an approach allows ORR to proceed adjacent to surface-bound ORR intermediate (OOH*, O*, and OH*) species if it is favorable. By considering the integration of these factors (termed the integrated approach versus the earlier independent site analysis), we show that DFT predicts high thermodynamic limiting potentials for pyridinic sites and lower thermodynamic limiting potentials for the majority of quaternary sites except for the basal quaternary sites.

## Computational Methods

### DFT Calculation Details

All DFT calculations were performed using the Vienna Ab initio Simulation Package (VASP)^43–46^ with the projector augmented wave (PAW)^47,48^ pseudopotentials provided in the VASP database. Exchange and correlation effects were described with the Perdew–Burke–Ernzerhof (PBE) exchange-correlation^49,50^ form of the generalized gradient approximation (GGA) functional. All calculations were carried out with spin polarization and with a plane-wave cutoff of 400 eV. The total energy calculations were accelerated using a Fermi-level smearing width of 0.1 eV, and dispersion interactions were incorporated through the D3 implementation (referred to subsequently as DFT-D3) by Grimme and co-workers.^51^ Within the DFT-D3 method, the dispersion forces and energy are obtained from an empirical expression and added to the conventional DFT forces and energy. All atoms were allowed to relax until the forces were less than 0.03 eV/Å. We employed VASPsol^52,53^ to include solvation effects using the default for the dielectric constant (ε_b_ = 78.4), width of the dielectric cavity (σ = 0.6), cutoff charge density (ρ_cut_ = 0.0025 Å^–3^), and surface tension parameter (τ = 0.525 meV/Å^2^).

The CN_*x*_ catalyst has a heterogeneous structure with several potential local structures characterized by the nature of the N species (pyridinic, quaternary, and pyrrolic), oxidation of the N-edge species (pyridinic oxide), and edge type (zigzag or armchair). This provides 12 different site models as shown in Figure 1 to represent the heterogeneous CN_*x*_ structure.^54^ Figure 1 also shows the abbreviation labels for the 12 sites that will be used throughout the paper. Basal planes, zigzag, armchair, and pyrrolic sites are sampled using 4 × 4 × 1, 4 × 1 × 1, 1 × 4 × 1, and 1 × 4 × 1 *k*-point meshes, respectively. The choice of k-point mesh is based on convergence of free energy of intermediates with higher k-point mesh (corresponding 8 × 8 × 1, 8 × 1 × 1, 1 × 8 × 1, and 1 × 8 × 1) within 0.03 eV.

**Figure 1:**
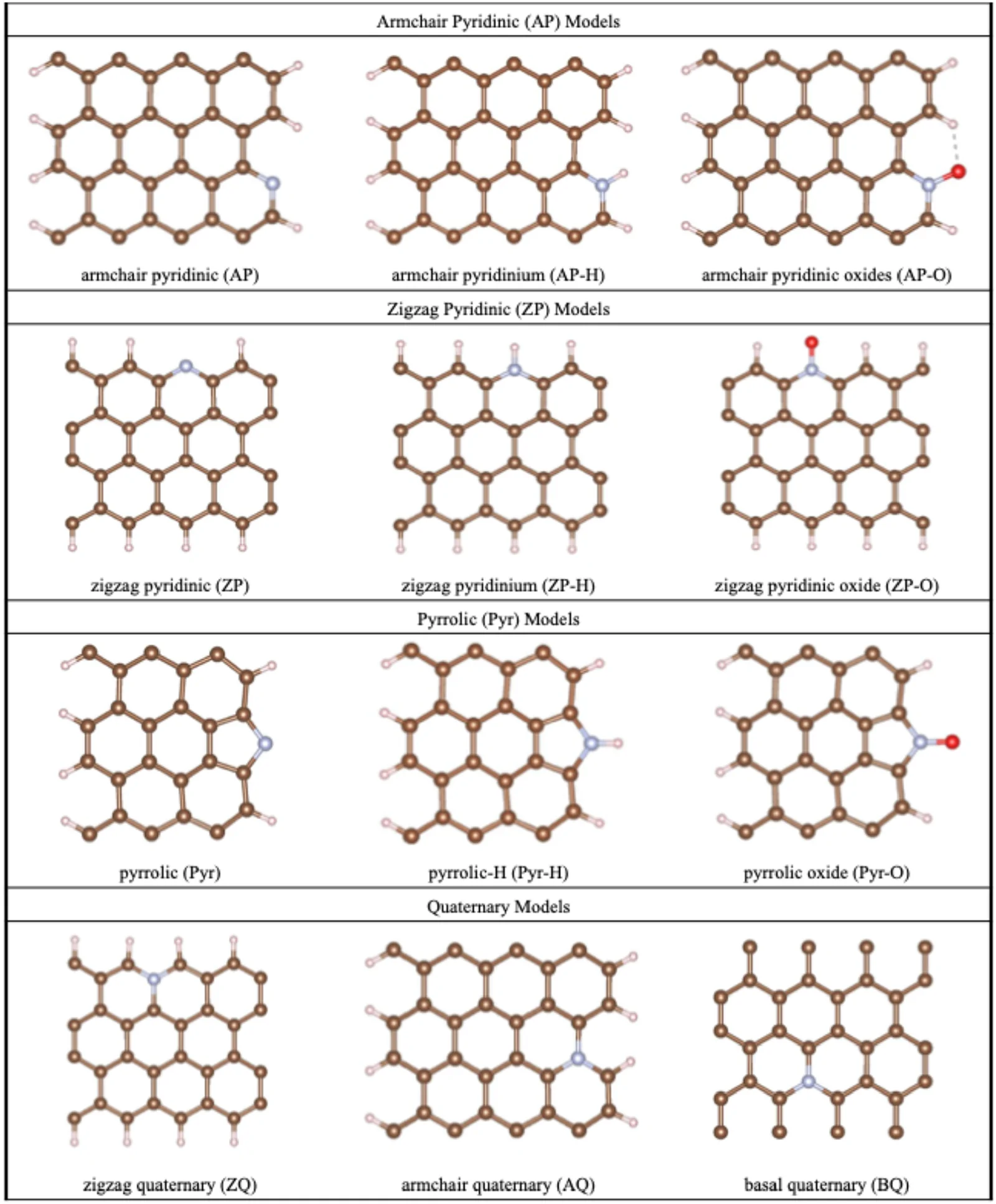
Computational CN_*x*_ models representing different active sites. Brown (carbon), silver (nitrogen), red (oxygen), and white (hydrogen).

### Potential-Dependent Free Energy of Elementary Steps

The computational hydrogen electrode (CHE) or linear free energy method allows for the calculation of the proton–electron pair free energy at varying potentials since the free energy of the proton–electron pair is equal to half the free energy of a single H_2_ molecule at the potential of the reference reversible hydrogen electrode (RHE), 0 V-RHE.^55–61^ All steps involving an electron are therefore shifted in free energy by −*eU*_RHE_, where *U*_RHE_ is the applied potential in V-RHE. The free energy of the proton–electron pair at any potential is given by eq 1, where the free energy of the electron varies linearly with applied potential.

1 $$G\left(H^{+}\left(aq\right)+e^{-}\right)=\frac{1}{2}G\left(H_{2}\left(g\right)\right)-eU_{RHE}$$

Equation 1 can then be combined with any elementary step involving a proton–electron transfer step. The general cathodic proton–electron transfer step for a surface species (A*) is given by

2 $$A^{*}+H^{+}\left(aq\right)+e^{-}\to AH^{*}$$

The free energy of this elementary step is given by eq 3 below

3 $$\Delta G_{rxn}\left(U\right)=G_{AH^{*}}-G_{A^{*}}-\frac{1}{2}G\left(H_{2}\left(g\right)\right)+eU_{RHE}$$

Similarly, the general anodic proton–electron transfer step is the reverse of eq 2 and given by

4 $${AH}^{*}\to A^{*}+H^{+}\left(aq\right)+e^{-}$$

The free energy of reaction for the above reaction is given by eq 5

5 $$\Delta G_{\mathrm{rxn}}\left(U\right)=G_{A^{*}}-G_{AH^{*}}+\frac{1}{2}G\left(H_{2}\left(g\right)\right)-eU_{RHE}$$

The mechanisms for ORR proceed via cathodic proton–electron transfer steps, with OOH*, OH*, and O* being the key ORR intermediates. For each intermediate (OOH*, OH*, O*) and any relevant coadsorbate configurations, the site minimization procedure is carried out as described in Section S1. The free energies of these ORR intermediates are determined as a function of the potential and following the convention in the literature referenced to the final state of the ORR (two H_2_O­(aq) molecules and the bare surface).^62^ The free energy of adsorbed species incorporates the vibrational free energy corrections using the vibrational frequencies obtained by fixing the CN_*x*_ structure. The free energies of H_2_O (aq) and H_2_ (g) are obtained from the DFT free energy calculations of these molecules at *T* = 298 K and saturation pressure for H_2_O (*P*_sat_ = 0.0313 atm) and standard pressure of 1 atm for hydrogen. Due to the well-known DFT accuracy issues for evaluating the isolated O_2_ (g) energy,^63,64^ the experimental standard free energy of reaction (Δ*G*_rxn_^o^ = 2.46 eV per water molecule) for the water splitting reaction $\left(H_{2}O\left(l\right)\to \frac{1}{2}O_{2}\left(g\right)+H_{2}\left(g\right)\right)$ is used along with the DFT free energies of hydrogen and water to obtain the O_2_ (g) free energy that satisfies Δ*G*_rxn_^o^ = 2.46 eV.^62,64^ The O_2_(g) is assumed to be in equilibrium with O_2_(aq) and thus the chemical potentials (or partial molar free energies) of O_2_(aq) are equal to O_2_(g).

The DFT ORR activity is then calculated in terms of the thermodynamic limiting potential. The DFT thermodynamic limiting potential for ORR is defined as the largest applied potential when all elementary steps are downhill or thermoneutral in free energy.^62^ The potential-determining step (PDS) is determined as the elementary step with the most positive free energy change at 1.23 V-RHE,^65^ which will be associated with the thermodynamic limiting potential. The onset potential will depend on potential-dependent barriers of elementary steps and not the free energy of reaction. However, for proton–electron transfer steps, it is expected that the barriers would be around 0.3–0.5 eV larger than the free energy of reaction, and thus, when a step is thermoneutral, the barrier could be overcome at room temperature.^66–68^ Following previous studies,^28,37,69,70^ we use the CHE method to evaluate dissociative mechanisms on ORR, similar to associative mechanisms despite there being a bond breaking in the first step. Therefore, we can take the DFT-CHE thermodynamic limiting potential as an upper bound for the onset potential (or a best-case scenario) because of the approximation of using the free energy of reaction instead of barriers. To fully incorporate features such as constant potential and evaluation of free-energy barriers^71–75^ would require more complex and computationally demanding calculations. In this paper, we focus on a range of active site models along with incorporation of more complex reaction pathways that we refer to as an integrated reaction mechanism. The computational tractability of the DFT-CHE approach allows for the systematic exploration of these features that have not been examined on CN_*x*_ catalysts, and we leave it to future work to incorporate more sophisticated electrochemical modeling approaches.

## Results and Discussion

### ORR Analysis on Independent CN_*x*_ Site Models

We begin our analysis by considering the different ORR mechanisms (Scheme 1) for each of the possible active CN_*x*_ sites shown in Figure 1. In this approach, each of the active site models for CN_*x*_ is treated as independent and potentially present in the experimental CN_*x*_ catalyst (referred to as the independent site analysis). As noted in the introduction, this is the common approach in the literature to the study of ORR on CN_*x*_ catalysts to identify potential active sites. We note that the mechanisms in Scheme 1 proceed via proton–electron transfer steps. Alternative mechanisms such as nonelectrochemical O_2_ dissociation or the sequential electron–proton transfer step (i.e., electron transfer to form O_2_^–^ and then proton transfer to form OOH*) are not included. Within the CHE approach, such steps would be intermediate and would result in the final steps shown in Scheme 1. Such intermediate steps could be impactful if they are the rate-limiting (or potential-determining) steps but would require other approaches such as constant potential grand canonical DFT and the evaluation of barriers and even explicit water molecules. Several experimental studies show no detectable H_2_O_2_ formation for CN_*x*_ catalysts via rotating ring-disk electrode (RRDE) experiments.^38,76,77^ Therefore, the H_2_O_2_ formation mechanism is not considered.

**Scheme 1:**
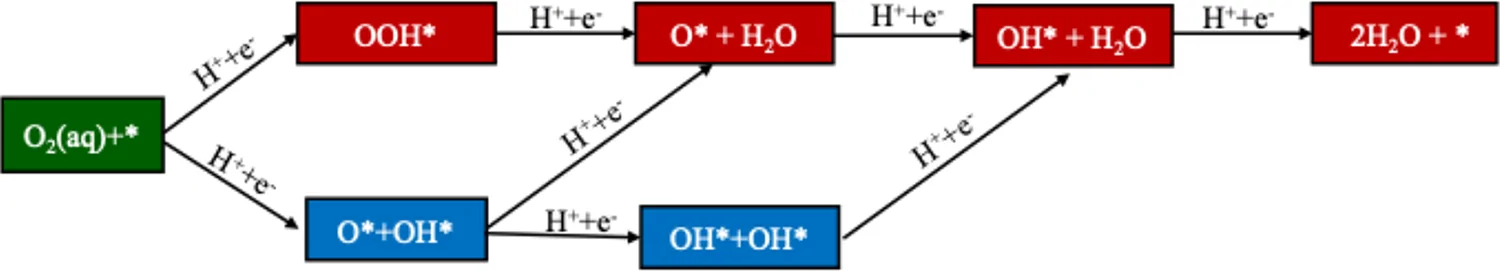
ORR Mechanisms in Acidic Medium Incorporating the Associative (Red) and Dissociative (Blue) Mechanisms

In Scheme 1, the dissociative mechanism combines with the associative mechanism later in the pathway. In these mechanisms, we do not consider the possibility of any of the intermediates not proceeding forward and a second O_2_ to initiate reaction on an adjacent site. Such a possibility can be termed as a “coverage” or neighbor-mediated mechanism. We have examined this effect for the bromine evolution reaction (BER) and found that BER proceeds through reactions occurring adjacent to an OH*.^78^ To our knowledge, such neighbor-mediated mechanisms have not been examined for ORR on CN_*x*_ catalysts. We will revisit such additional complexity in the mechanisms later in the paper.

Figure 2 shows the comparison of the thermodynamic limiting potential for different CN_*x*_ models using associative and dissociative mechanisms. For the dissociated state (O* + OH*), we find that protonation of the OH* over the O* is favored for all CN_*x*_ models (Section S2, Table S5). BQ under the dissociative mechanism shows the peak DFT activity with a thermodynamic limiting potential of 0.99 V-RHE. To aid in the discussion, we term models within 75% of this thermodynamic limiting potential (0.75 to 0.99 V-RHE) to fall into the group with higher activity. The PDS for different models, along with corresponding thermodynamic limiting potentials for these mechanisms, are shown in Section S3, Table S6.

**Figure 2:**
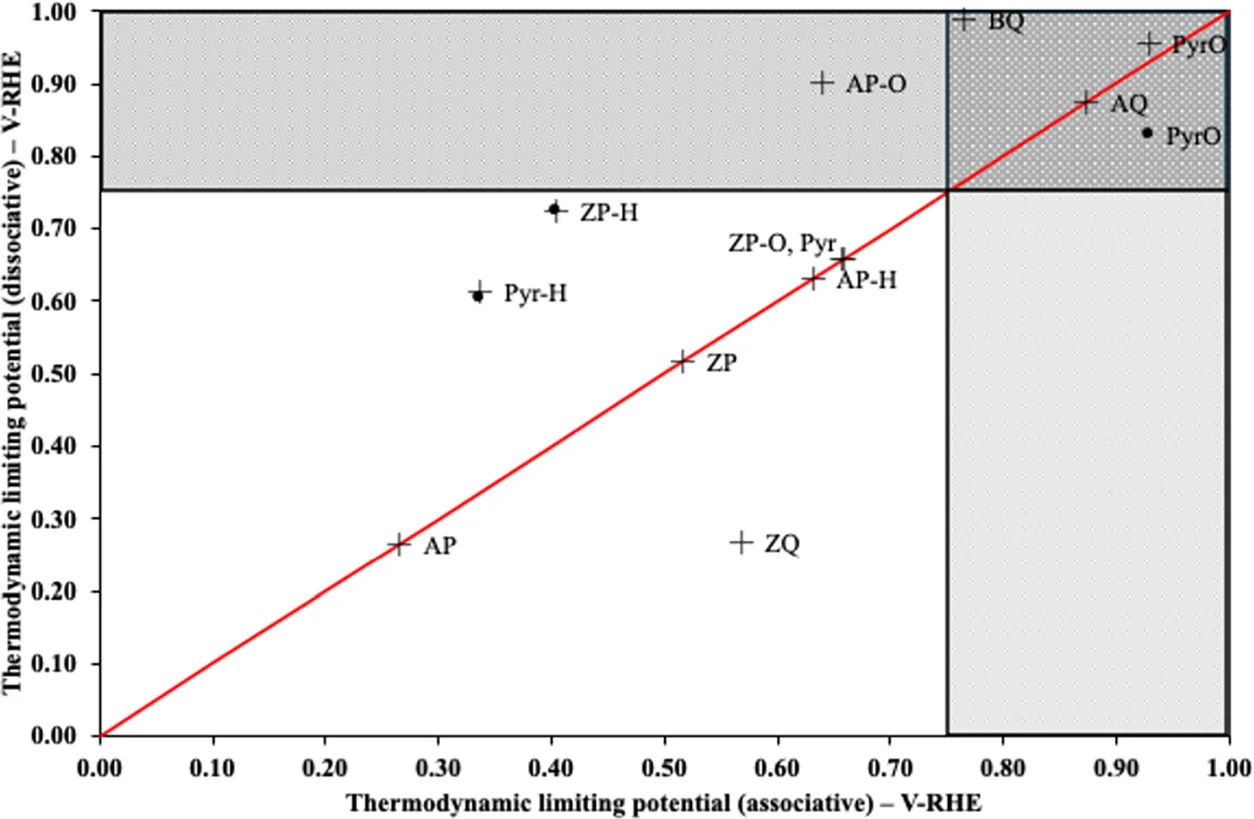
DFT-predicted ORR thermodynamic limiting potentials for CN_*x*_ site models (see Figure 1) via associative and dissociative mechanisms (pH = 0). The gray region highlights site models with thermodynamic limiting potentials greater than 0.75 V–RHE.

In Figure 2, several models overlap in thermodynamic limiting potential, indicating that the PDS is common to both the associative and dissociative mechanisms. For these cases, the PDS would be late in the mechanism, and we find that all these cases except for AP involve OH* → H_2_O as the PDS. AP is a special case where the thermodynamic limiting potential overlaps but in fact the associative and dissociative mechanisms have different PDS. When undergoing the associative mechanism, the PDS is either O_2_ → OOH* or OH* → H_2_O. This is indicated by the binding strength of OH*. Models with weak OH* binding models have PDS of O_2_→OOH* while the models with strong OH* binding models have PDS of OH* → H_2_O. The trend is less clear for the dissociative mechanism. The strong OH* binding models predominantly prefer to have PDS of OH* → H_2_O, except for ZQ, which has PDS of O* + OH* → O*. The weak binding models generally prefer to have PDS of O_2_ → O* + OH*; however, ZP-H and Pyr-H have PDS of O* → OH*. Thus, when considering the dissociative mechanism, four types of PDS can take place.

AQ, BQ, and PyrO are active when considering either associative or dissociative mechanisms, while AP-O is active only via the dissociative mechanism. Thus, predominantly quaternary/oxide sites show high activity. This is in good agreement with previous DFT studies which show high activity for quaternary and pyridinic oxide sites.^11,33,34,36,39,79^ Liang et al. examined ORR on CN_*x*_ with several N-doped structures, finding armchair graphitic-N (AQ) to have peak activity along with several oxidized N-doped structures to be active sites.^36^ Wang et al.^34^ and Zhang et al.^11^ reported that graphitic-N (quaternary species) has higher activity than pyridinic species considering single-doped N-species. Further, from Figure 2, the three pyridinic sites (ZP/AP/Pyr) all show relatively low thermodynamic limiting potential (<0.7 V-RHE) and thus lower activity. Thus, our results are in good agreement with previous DFT papers that applied the CHE method to independent site analysis. However, these DFT results do not show good agreement with earlier experimental papers, indicating that pyridinic sites are the marker of ORR active sites.^14,23–25,38^

### Surface Stability of CN_*x*_ Sites before the Start of ORR

In the approach above, the ORR mechanisms are evaluated based on the independent site model analysis. However, the catalyst surface can undergo changes before ORR is even initiated. In particular, changes to the N-edge sites of the CN_*x*_ catalyst can occur through protonation and water oxidation steps in the absence of molecular oxygen via the reactions shown below, where X represents an N-edge site (X = ZP/AP/Pyr) of CN_*x*_.

6 $$X+H^{+}+e^{-}\to X-H$$

7 $$X+H_{2}O\to X-OH+H^{+}+e^{-}$$

8 $$X-OH\to X-O+H^{+}+e^{-}$$

Reaction 6 involves a cathodic proton–electron transfer step, while reactions 7 and 8 are anodic steps where the water molecule provides the O atom. Reactions 7 and 8 are normally associated with oxygen evolution via water oxidation, which occurs above 1.23 V-RHE. Nevertheless, these intermediate steps can occur at 1.23 V-RHE and below. However, with increasing cathodic applied potential, the anodic steps of (7) and (8) will become unfavorable and the reverse reactions will occur corresponding to ORR. In this section, our interest is on the changes to CN_*x*_ at 1.23 V-RHE before the start of ORR (introduction of bubbling O_2_ in the experiment), and thus reactions 6–8 are the relevant reactions to consider.

Although eqs 6 to 8 involve oxidation of edge N atoms, other similar reaction steps can occur that involve the formation of O* or OH* on carbon atoms adjacent to the nitrogen atom. For example, reactions 7 and 8 can occur sequentially to form ZP/O*, denoting oxygen formation on the carbon atom adjacent to the N dopant. There are many combinations that can occur to yield a range of possible structures that can form from the original edge N-doped structure. This is illustrated for the case of ZP in Figure 3, where possible structures are classified by changes to the N site. Similar structures hold for the AP and Pyr edge sites (not shown). We note that one could continue to oxidize these structures (e.g., ZP-O/2O*); however, we find that the free energy changes to form such higher oxidized structures are unfavored at 1.23 V-RHE for all N-dopant structures considered in this study.

**Figure 3:**
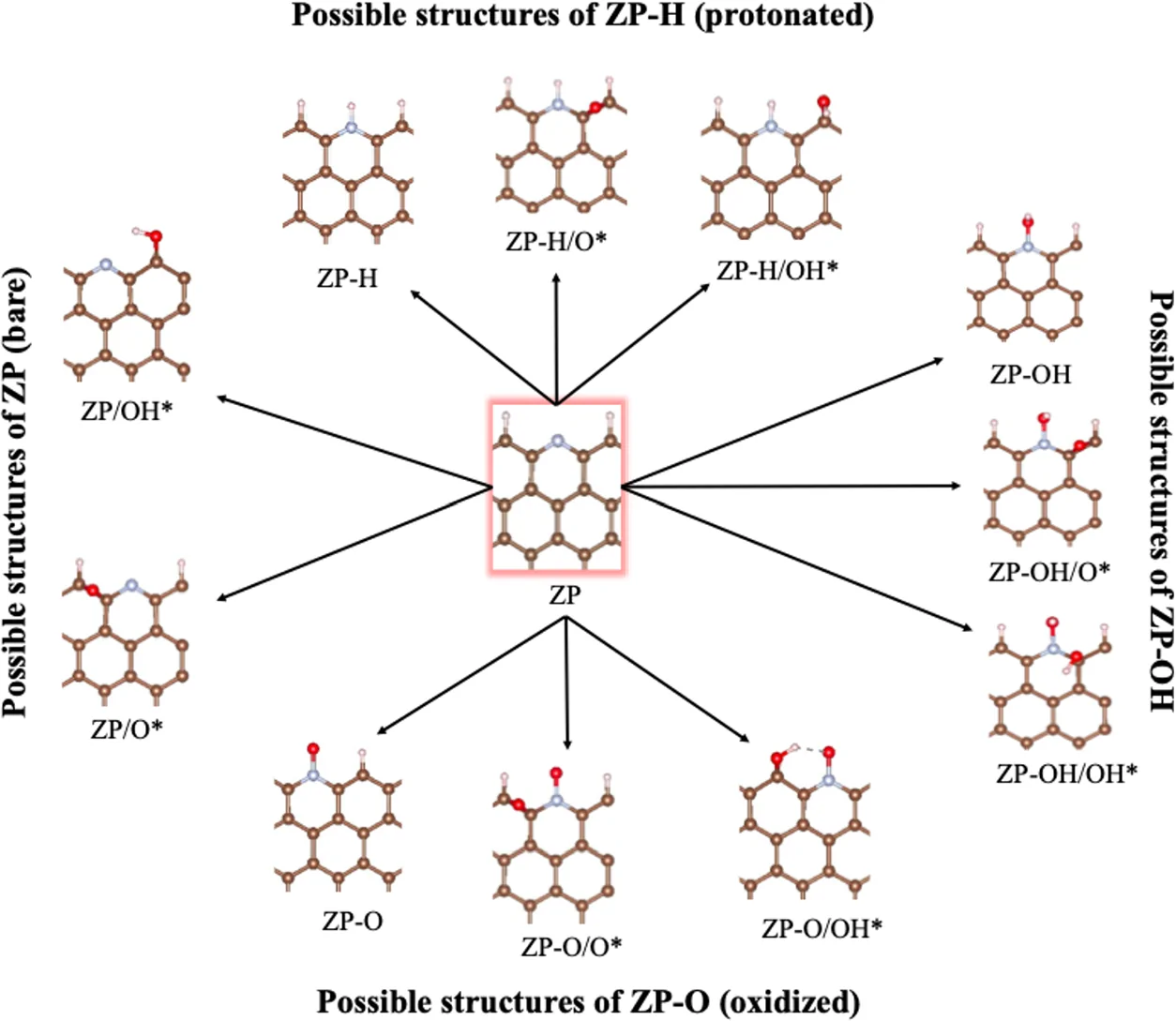
Possible structures accessible from the zigzag pyridinic (ZP) site via protonation and water oxidation in the absence of molecular oxygen prior to ORR.

To examine the various structures in Figure 3, we need to evaluate the free energy with respect to a common reference. Taking this reference as the bare structure (e.g., ZP), we can incorporate all the changes due to protonation/oxidation with eq 9 below.

9 $$X+aH_{2}O+b\left(H^{+}+e^{-}\right)\to Y$$

where X is the initial bare N-dopant structure and Y represents the corresponding protonated/oxidized structure formed. Table 1 shows the list of protonated/oxidized structures considered in this study and the corresponding integer values of *a* and *b* in eq 9. The values for *a* and *b* are obtained from the sum of the elementary steps in eqs 6–8. For reaction 6, *a* = 0 and *b* = 1 and for reaction ([Disp-formula eq7]), *a* = 1 and *b* = −1 and for reaction ([Disp-formula eq8]), *a* = 0 and *b* = −1. For instance, to form ZP–OH from ZP involves one H_2_O molecule (*a* = 1) with a corresponding anodic proton–electron transfer (*b* = −1). We have evaluated the free energy for all the reactions outlined in Table 1 for the pyridinic and quaternary structures and the data is reported in Table S7 in Section S4.

**Table 1:**
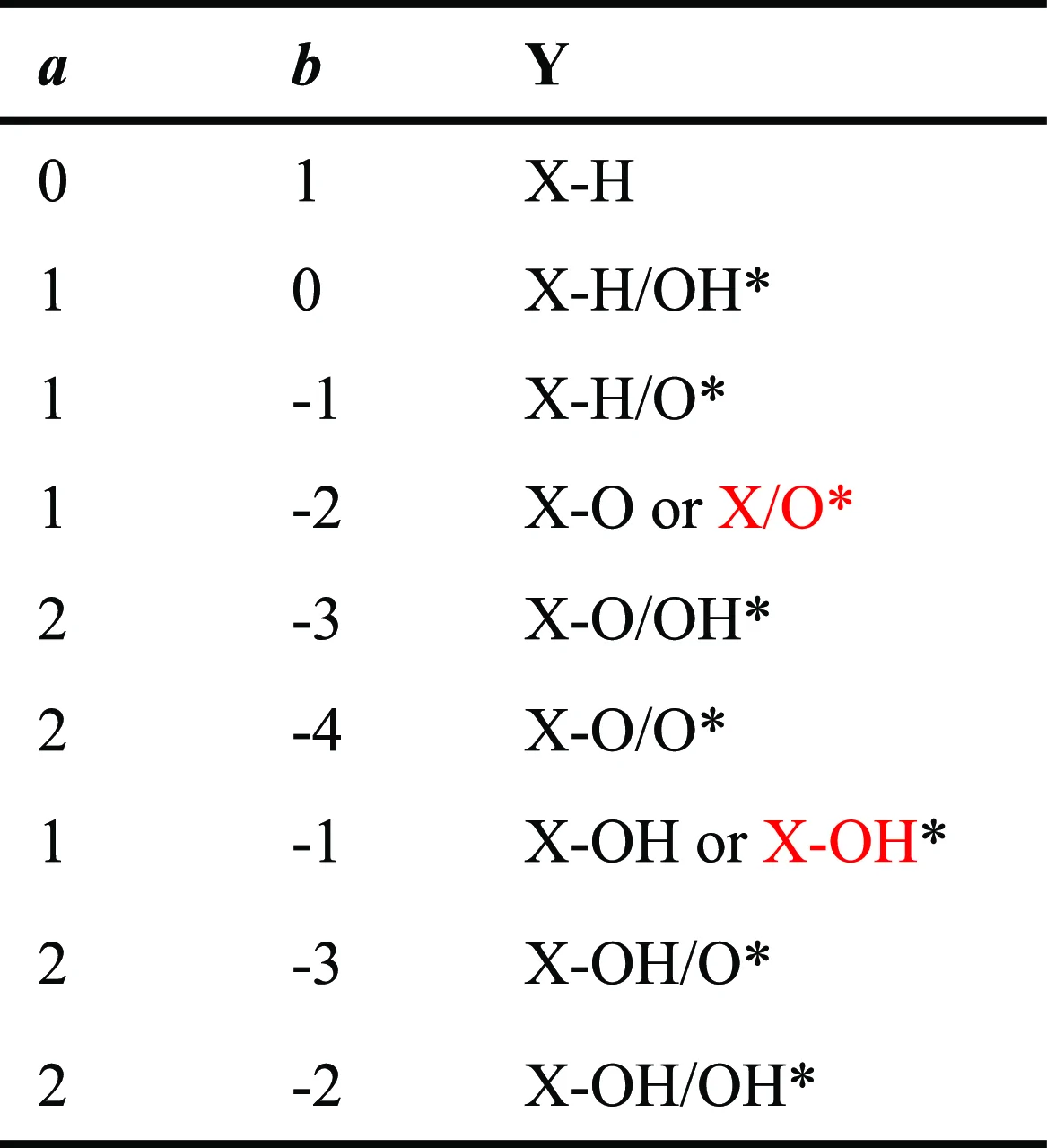
Values of *a* and *b* in eq 9 Used to Generate Different Structures (Y) in the Absence of Molecular Oxygen from X (N-dopant)^a^

a Note: Quaternary N cannot form direct bonds with H, O, OH, so are constrained to a subset of only two of these structures highlighted in red.

We first consider the case for ZP with the resulting surface Pourbaix diagram shown in Figure 4. Through the surface Pourbaix diagram, we can determine the most stable structure as a function of applied potential. Thus, at the beginning of ORR (1.23 V-RHE), ZP-O/OH* is thermodynamically preferred and this is the case until ∼0.85 V-RHE, after which ZP-H becomes more stable. This span of potentials that favor ZP-O/OH* suggests that even with the caveat due to the simplifications in the CHE DFT approach, we can expect ZP structures to oxidize to ZP-O/OH* before the start of ORR. This result stands in contrast to the independent analysis approach results presented in Figure 2, where ZP and ZP-O were considered as independent sites. Here, the prediction is that any ORR activity associated with ZP sites would in fact be due to the formation of ZP-O structures.

**Figure 4:**
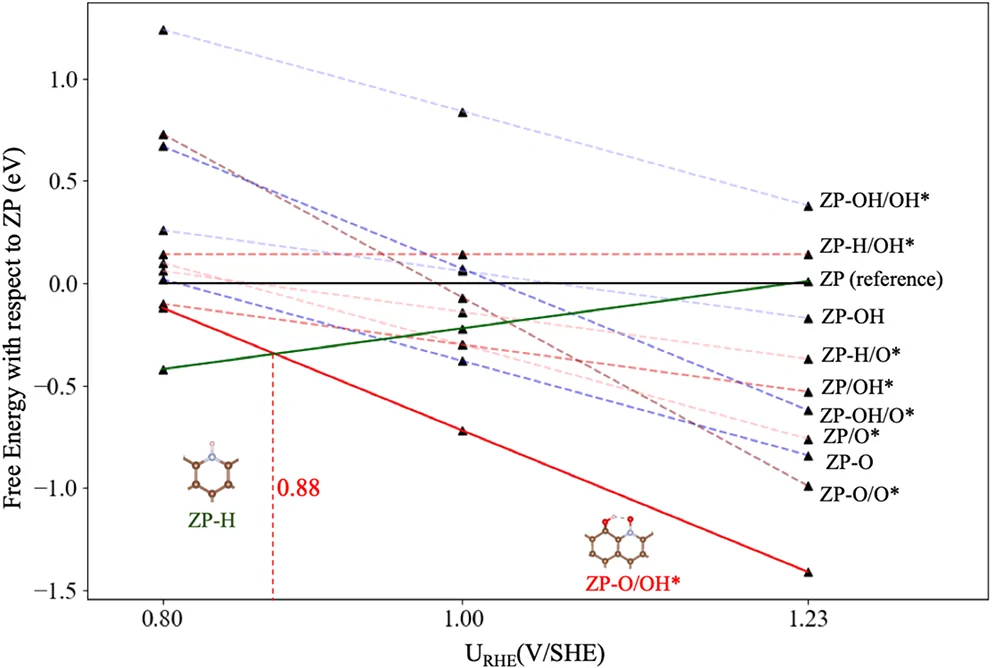
Surface Pourbaix diagram for zigzag pyridinic (ZP) site at pH = 0 in the absence of molecular oxygen showing the relative stability of different structures as a function of applied potential.

The formation of ZP-O/OH* from the bare ZP structure however involves a series of elementary steps which may involve uphill steps. One such sequence of elementary steps would be ZP → ZP–OH → ZP-O → ZP-O/OH*. As can be seen in Figure 4, the free energy change for all these steps is negative at 1.23 V-RHE and thus, with the assumption that the barriers associated with proton–electron transfer steps are in the range of 0.3–0.5 eV for Δ*G* ≤ 0, DFT does not predict any issues in accessing the thermodynamically preferred state for ZP. Thus, through this pathway, ZP-O/OH* is still preferable. An alternate sequence of pathways exists via ZP → ZP/OH* → ZP–OH/OH* → ZP-O/OH* to get the same final state. Through this route, ZP/OH* → ZP–OH/OH* is uphill with a free energy change of 0.92 eV, indicating that a kinetic barrier would be encountered along this path.

The surface Pourbaix diagram for AP is shown in Figure 5. Similar to ZP, the thermodynamically preferred structure for AP is AP-O/OH* until the cathodic potential reaches ∼1.05 V-RHE, where the AP edge site becomes favored. However, unlike ZP, most of the intermediate structures in the transition from AP to AP-O/OH* are above the reference state for AP over a large range of potentials. Due to this, in the case of AP, we expect significant uphill steps when going from AP to AP-O/OH*. First, considering AP → AP–OH → AP-O → AP-O/OH*, the free energy change for the first step going from AP→ AP–OH is 1.15 eV. This indicates a large barrier for AP going along this route and subsequent conversion to AP-O/OH*. However, for an alternate route, AP → AP/OH* → AP–OH/OH* → AP-O/OH*, we find a smaller but still positive free energy change of 0.38 eV for the first step. Thus, DFT-CHE predictions suggest that AP is more difficult to oxidize than the other pyridinic edge sites and at 1.23 V-RHE (start of ORR) the AP site might be present instead of an oxidized AP edge site. However, for our analysis of ORR in the next section, we will retain the thermodynamically predicted AP-O/OH* as the starting point and revisit the implications on the ORR reactivity of AP for both cases (oxidized vs retaining the bare AP structure).

**Figure 5:**
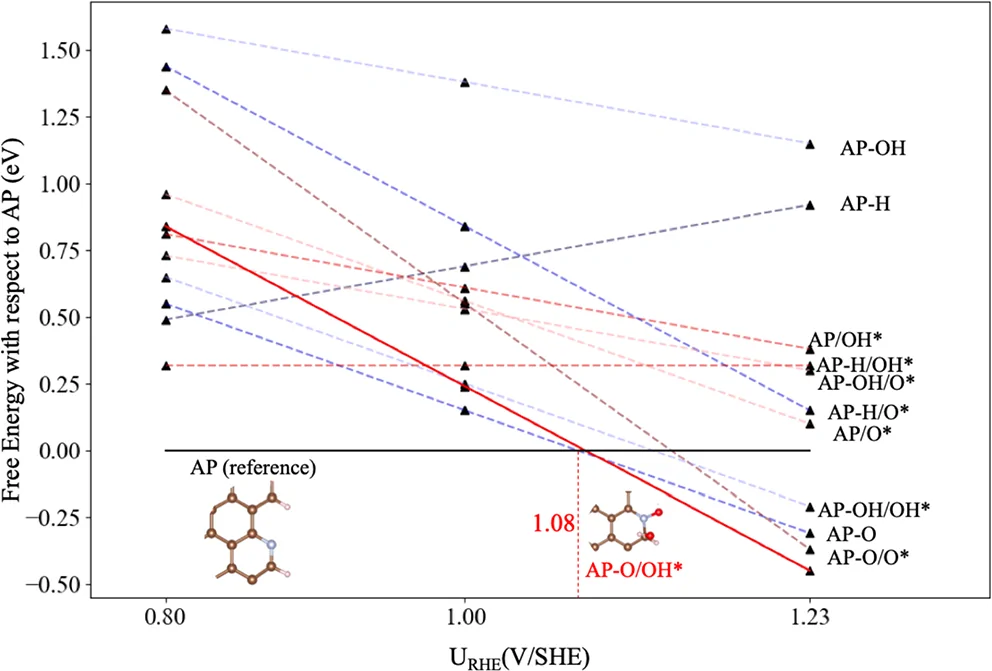
Surface Pourbaix diagram for armchair pyridinic (AP) site at pH = 0 in the absence of molecular oxygen showing the relative stability of different structures as a function of applied potential.

Finally, the surface Pourbaix diagram for the Pyr edge is shown in Figure 6. The Pyr edge is similar to ZP in that the Pyr-O/OH* is thermodynamically favored, and the intermediate steps also show negative free energies of reaction at 1.23 V-RHE. Thus, we find that for all the edge sites (X = ZP, AP, and Pyr), the thermodynamically most stable structure is X-O/OH*, with ZP and Pyr showing elementary steps that will be accessible at 1.23 V-RHE provided there are no significant additional barriers associated with the proton–electron transfer step. However, AP shows much less preference for oxidation, with several intermediate steps being uphill at 1.23 V-RHE.

**Figure 6:**
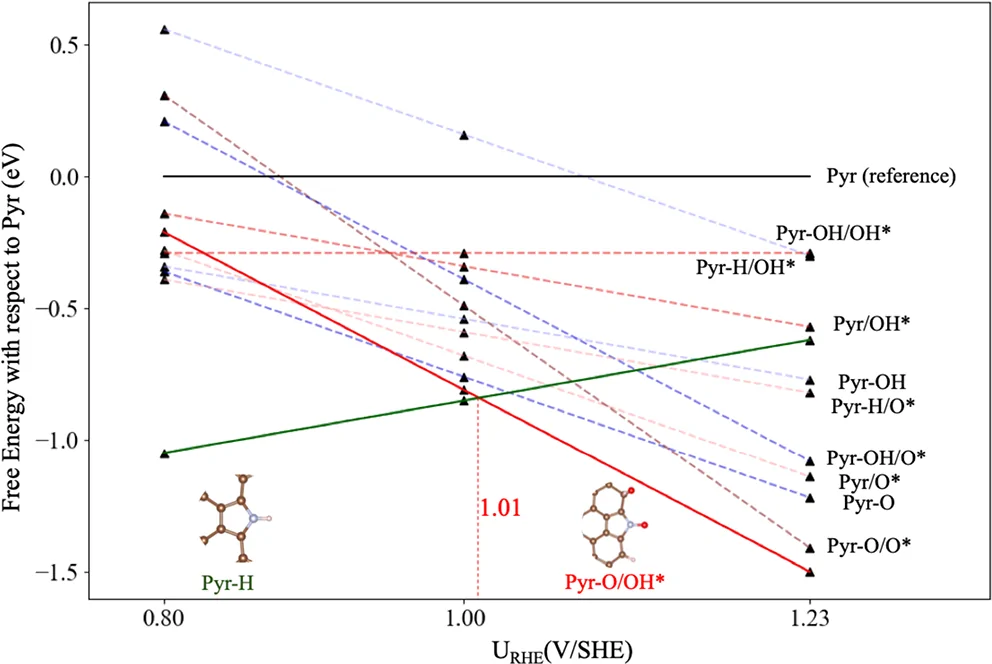
Surface Pourbaix diagram for pyrrolic (Pyr) site at pH = 0 in the absence of molecular oxygen showing the relative stability of different structures as a function of applied potential.

In the case of quaternary sites (X’ = ZQ, AQ, BQ), the direct protonation/oxidation of the nitrogen site is not possible due to nitrogen being bonded to 3 carbon atoms. Thus, the only possible protonated/oxidized structures are those involving adsorption of oxygen (O*) or hydroxyl (OH*) on the carbon atoms nearby to the nitrogen dopant. For all cases, the most stable structure at 1.23 V-RHE is X’/O* (Section S4, Table S7). Considering the elementary steps leading to X′/O*, the only possible pathway is X′ → X′/OH* → X′/O* and all these steps are found to be downhill at 1.23 V-RHE, indicating the thermodynamic X’/O* structure should be accessible at the start of ORR for the quaternary sites.

### Integrated ORR Mechanism for N-Edge and Quaternary Models

After determining the most stable structure for the CN_*x*_ structure at 1.23 V-RHE, we now consider the ORR activity of each of these structures using an integrated ORR mechanism. We refer to this approach as an integrated ORR mechanism because we consider (1) the elementary steps of ORR from the O_2_ molecule, (2) protonation steps (including those that can occur to change surface intermediates present prior to the start of ORR at 1.23 V-RHE), and (3) the possibility of additional ORR steps adjacent to ORR intermediates already on the CN_*x*_ surface. This last scenario is similar to the coverage effect that has been examined on metal surfaces, where instead of the initial oxygen species converting to water there is a buildup of oxygen species coverage (O*, OH*) before the ORR reaction begins to turn over.^27,80,81^ On CN_*x*_ the situation is different from that on a metal surface since the main active site is associated with the N atom and therefore, we only examine such additional ORR intermediates when a carbon atom directly adjacent to the N-dopant is empty. This rule allows us to limit the number of relevant configurations to examine. We have tested the effect of relaxing this rule on the predicted ORR integrated cycle for ZP and BQ and find that it does not modify the preferred steps in the pathway (see Section S7 in the SI for details). Thus, when considering the preferred pathway, we consider the competition between all the above-discussed steps and identify the elementary step with the minimal free energy change. Once this is evaluated, we then proceed along that pathway and examine the next step in the same manner. Once a sequence of steps that turn over the ORR is identified, we can determine the thermodynamic limiting potential associated with the PDS in the mechanism associated with the most positive Δ*G*. This procedure is easier to understand graphically, and the integrated ORR mechanism obtained for ZP is shown in Figure 7.

**Figure 7:**
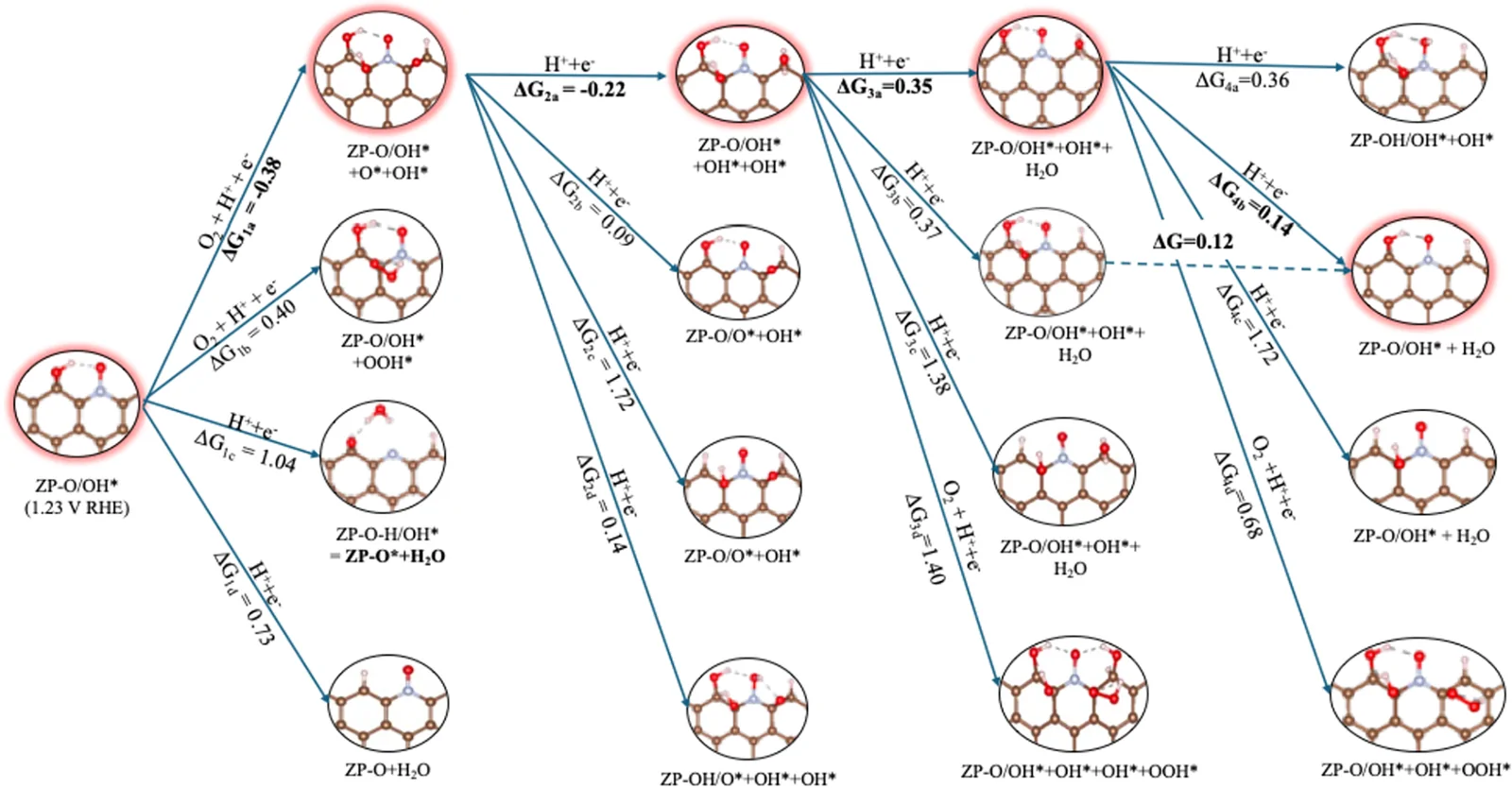
Protonation/ORR steps involved when considering integration of surface stability and ORR for ZP reported at 1.23 V-RHE. The free energies are reported in eV. The dashed arrow indicates an alternative pathway that is within 0.05 eV of the most preferred pathway.

Initially, the ZP structure is converted to the thermodynamically favored structure of ZP-O/OH* at 1.23 V-RHE (see Figure 4). At this point, oxygen is bubbled in the experiment, and the applied potential is changed in the cathodic direction (decrease in potential). We then considered all possible oxygen addition and protonation steps from this initial structure as the first step in the ORR mechanism. As shown in Figure 7, this includes O_2_ forming either dissociated O* + OH* or OOH* on the surface (the first step in Scheme 1) but also the possibility of protonation of the existing O species on the surface. The favored step (that is already negative at 1.23 V-RHE) is the formation of O* + OH* from O_2_, as shown in red in Figure 7. The next step (second) can involve protonation of four of the oxygen species (2OH*, O*, and ZP-O). We have not considered additional formation of O surface species from another O_2_ molecule here because the adjacent C atoms to the N dopant are occupied with O surface species. Here, the step that is favored is the protonation of the O*, leading to three OH* species on the surface. This is a configuration that cannot develop in the standard approach discussed earlier, but considering the integrated approach is favored even at 1.23 V-RHE. The next step (third) considers additional protonation of the OH* species, but now there is an available carbon site near the N dopant that is considered for another O_2_ molecule to react to form the OOH*. However, this is not favored and from Figure 7 the third step involves protonation of one of the OH* to form H_2_O. Subsequently, the last step is another protonation to produce H_2_O and results in the initial ZP-O/OH* structure.

In summary, there are four proton–electron transfer steps, with the first step being the O_2_ initial reaction. Overall, this is a relatively straightforward process, with the main distinction being the initial oxidation structure (ZP-O/OH*) that forms prior to ORR mediating the ORR steps. The third step in this mechanism has the largest positive Δ*G* (0.35 eV) and so would be the PDS and result in a thermodynamic limiting potential of 0.88 V-RHE. In addition, the third step has another alternative that is very close in energy (0.37 eV) but even proceeding in that direction would lead to a similar four proton–electron sequence as highlighted in Figure 7 by the dashed arrow. We applied this procedure for AP and Pyr edges and figures equivalent to Figure 7 for these edge sites can be found in Section S5.

We note that in Figure 4 the stability analysis of ZP edge shows that the ZP-O/OH* configuration is stable until ∼ 0.85 V-RHE. This stability limit of ZP-O/OH* is close to the predicted thermodynamic limiting potential of ZP from Figure 7, but it is important to stress that Figure 4 is derived under conditions of no O_2_. Therefore, Figure 4 is not directly relevant for the reaction conditions with flowing O_2_ outside of providing an initial structure at the start of the ORR experiment at 1.23 V-RHE. Instead, the integrated ORR mechanism approach highlighted in Figure 7 explores protonation steps that could remove the O species from ZP (i.e., convert ZP-O/OH* to ZP-O or ZP) but these are not favored over the preferred ORR mechanism. Within the CHE formulation, we are assuming that at a given applied potential, the ΔG that is most negative will dominate the selectivity of any branching point. So, for instance, at a 0.88 thermodynamic limiting potential, steps 2b and 2d would both have negative ΔG values; however, the preferred pathway through 2a would still have the most negative ΔG value and would be favored over the two alternate pathways. This may be a safe assumption for these steps since they all involve similar proton–electron transfer steps involving protonation of O surface species.

### ORR Integrated Cycle for Edge Sites

The detailed integrated mechanism analysis for AP and Pyr, similar to the analysis shown for ZP in Figure 7, are presented in Section S5 of the Supporting Information. Figure 8 shows the resulting net ORR cycle for ZP, AP, and Pyr edge sites obtained from the integrated ORR mechanism analysis. As discussed earlier, the independent site analysis predicts that the ZP, AP, and Pyr edge sites have lower thermodynamic limiting potentials of 0.52, 0.27, and 0.66 V-RHE, respectively. In contrast, as illustrated in Figure 8 the integrated mechanism shows a dramatic increase in the ORR activity of these edge sites with ZP, AP and Pyr edge sites now predicted to have thermodynamic limiting potentials of 0.88, 0.85, and 0.78 V-RHE. This increase in the thermodynamic limiting potential could be attributed to the increase in coverage of O and OH in the integrated mechanism as seen from Figure 8 in comparison to 4e^–^ ORR mechanism. Further, there is a significant change in the PDS. The independent site analysis mainly involves initial ORR intermediates like O*, OH*, OOH*, and O*+OH* in the PDS steps, as seen from Table S6, Section S3. Whereas in the case of the integrated mechanism, the PDS involves multiple O and OH species adsorbed on the surface, as can be seen from Figure 8. This shows that the neighbor-mediated mechanism plays an important role in ORR on CNx catalysts.

**Figure 8:**
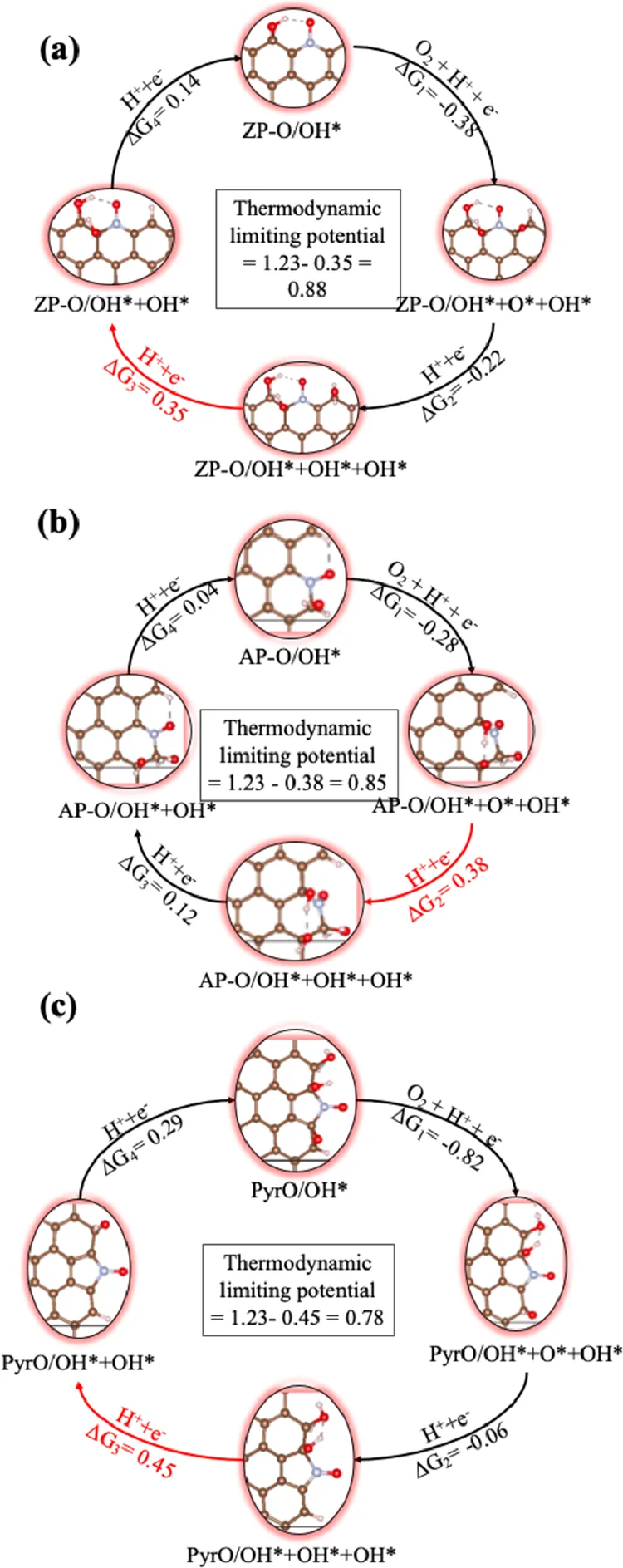
Preferred ORR cycle identified for (a) ZP, (b) AP, and (c) Pyr proceeds from ZP-O/OH*, AP-O/OH*, and PyrO/OH* (highlighted in blue), respectively. See Section S5 for the detailed integrated analysis for the pyridinic sites. Free energies and thermodynamic limiting potential are reported in eV and V-RHE, respectively. The PDS is highlighted in red.

Thus, from the integrated mechanism, all the pyridinic/pyrrolic models fall into the active region, which is in contrast to previous independent site model analysis that predicts these sites to be not active. In all these cases, the key feature is that the edge sites convert to an oxidized structure at 1.23 V-RHE that facilitates the ORR reaction. This result can reconcile the differences between experiments and DFT calculations as follows. In the experiment, the as-synthesized CN_*x*_ catalysts are characterized with XPS and the fractions of pyridinic, quaternary, and pyridinic oxide N in the sample are obtained. At the start of ORR (before any oxygen is bubbled), our DFT analysis predicts that the edge sites (pyridinic and pyrrolic) convert to an oxidized structure of X-O/OH*. This would also occur to any initially pyridinic oxide in the sample—i.e., all edge N atoms convert to this oxidized form whether they start as pyridinic or pyridinic oxide. Therefore, the experimental results that correlate pyridinic nitrogen to ORR activity would be valid, but the actual mechanism is revealed to be mediated through the X-O/OH* structure. Confirming this would require *in situ* XPS that could track changes to the edge N state during reaction. However, this is challenging and, to our knowledge, has not been done for CN_*x*_ catalysts.

In addition, as noted above, AP is not readily oxidized to the thermodynamic state with intermediate steps uphill at 1.23 V-RHE. We have performed an integrated ORR analysis on AP under the scenario that the thermodynamically oxidized state is inaccessible at 1.23 V-RHE. These results show that AP is inactive (see Section S5), and we conclude that the transition to the AP-O/OH* is critical to AP edge sites being ORR active. Interestingly, recent experiments have shown that operating in OER (1.5–1.6 V-RHE) before proceeding with the ORR sweep leads to higher activity.^82^ These experiments lend some support to our findings that the oxidized structures (AP-O/OH*) are precursors to the edge CN_*x*_ sites to be active for ORR. Under this interpretation, the potential held at 1.5–1.6 V-RHE is sufficient to overcome some of the intermediate steps to convert AP to the thermodynamically stable AP-O/OH* that then results in additional activity. Under these more positive potentials, corrosion and degradation of the carbon-based catalyst are concerns.^83,84^ Ozkan and co-workers have shown that CN_*x*_ can be resistant to carbon corrosion in contrast to Vulcan carbon or MeCN catalysts, but the stability is sensitive to the CN_*x*_ synthesis methods.^85,86^ However, to fully test the DFT predictions will require in situ XPS of CN_*x*_ during potential holds that vary applied potential and duration, and to our knowledge, such studies have not yet been reported.

### ORR Integrated Cycle for Quaternary Sites

We now use a similar integrated mechanism analysis to generate the net ORR cycle for quaternary sites (ZQ/AQ/BQ) shown in Figure 9. Section S6 shows the detailed integrated analysis for all the quaternary sites. In contrast to edge sites, the integrated mechanism for zigzag and armchair quaternary sites involves six steps, with formation of a cycle after the first two steps. After formation of the most stable structure, X′/O* at 1.23 V-RHE, the subsequent first two steps lead to formation of X′/O*+OH*+OH*. After these initiation steps, an overall cycle is formed, leading back to the formation of X′/O*+OH*+OH*. The thermodynamic limiting potentials observed for ZQ and AQ through this integrated mechanism are 0.53 and 0.57 V-RHE, respectively. In comparison, the thermodynamic limiting potentials from the independent site analysis for ZQ and AQ are 0.57 and 0.87 V-RHE, respectively. Thus, AQ goes from a high active site in the independent site analysis to a low active site once the integrated mechanism is applied.

**Figure 9:**
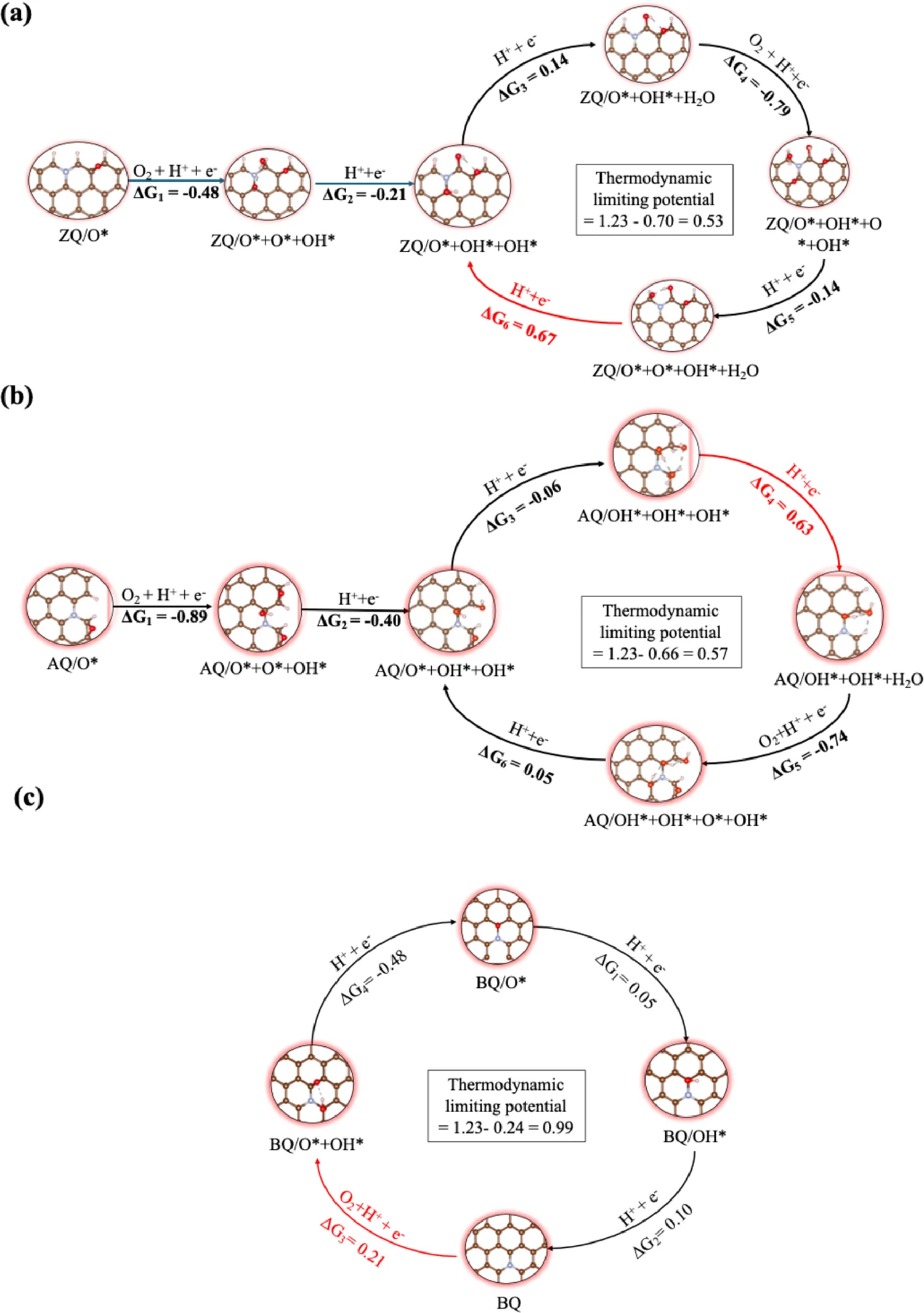
ORR cycle for (a) ZQ, (b) AQ, and (c) BQ proceeds from ZQ/O*, AQ/O*, and BQ/O*, respectively. See Section S6 for the detailed integrated analysis for the quaternary sites. Free energies and thermodynamic limiting potential are reported in eV and V-RHE, respectively.

In the case of BQ, the integrated mechanism starts with formation of BQ/O* but then follows a similar mechanistic route as the dissociative ORR mechanism found in the independent site analysis. Thus, the PDS and thermodynamic limiting potential identified through the integrated mechanism are O_2_ → O* + OH* and 0.99 V-RHE, respectively, exactly the same as in the independent site analysis.

From our discussion, the integrated ORR mechanism shows that edge models have high activity, while quaternary models show low activity in most cases. Although BQ shows high activity, the XPS binding energy for BQ is not readily distinguishable from those of other quaternary sites (ZQ and AQ).^54,87^ One plausible explanation of the experimental studies that correlate activity with pyridinic sites over quaternary sites is that the synthesized CN_*x*_ catalysts are dominated by edge sites (or defects in the basal plane), and therefore, the quaternary sites would be dominated by AQ/ZQ. There is some support for this interpretation since the CN_*x*_ catalysts are synthesized using metal precursors (Fe, Co) that initially lead to MeCN structures.^88^ They are then acid-washed to remove the metals, leading to CN_*x*_ edge structures. Such a process is less likely to produce doped N on a basal graphene plane, and quaternary N may be predominantly associated with edge sites (i.e., AQ/ZQ). Experimental studies that produce a high density of basal quaternary sites would be needed to further clarify the role of basal quaternary on ORR activity under acidic conditions.

## Conclusions

In summary, we examined the ORR activity of CN_*x*_ catalysts using DFT and an integrated framework that (1) incorporates stability analysis of the N-dopant site models at 1.23 V-RHE, prior to the start of ORR, via a surface Pourbaix analysis (pH = 0) and (2) accounts for alternative ORR steps adjacent to pre-existing surface species. To establish a baseline for our comparison, we first study the ORR activity on 12 different CN_*x*_ site models representing a range of quaternary and pyridinic structures, treating each site model independently. The independent site analysis predicts high activity for the quaternary/oxide sites and low activity for the pyridinic sites, which agrees with previous DFT papers but contradicts experimental studies that correlate pyridinic sites to ORR activity. We then considered the surface stability of CN_*x*_ sites in the absence of molecular oxygen, evaluating the relative stability of bare, protonated, and oxidized structures to generate a surface Pourbaix diagram. From the surface Pourbaix diagram, the most stable structure for pyridinic sites (X = ZP/AP/Pyr) is XO/OH* and for quaternary sites (X′ = ZQ/AQ/BQ) is X′/O* at 1.23 V-RHE. Using these initial thermodynamically preferred pyridinic/quaternary structures, an integrated ORR mechanism was examined to develop a four proton–electron catalytic cycle for ORR and an associated thermodynamic limiting potential. The resulting thermodynamic limiting potentials are high for pyridinic sites, indicating enhanced ORR activity, whereas quaternary sites—except for basal quaternary—exhibit lower activity. Based on these results, we conjecture that the experimental correlation between pyridinic sites and ORR activity can be attributed to the initial formation of pyridinic oxide sites that facilitate the higher ORR activity. This work shows that DFT predictions of the active sites for CN_*x*_ can be reconciled with experiments if changes to the nature of the active site under reaction conditions are incorporated in the modeling.

## Supplementary Material



## Notes

Site minimization procedure; evaluation of competition between elementary steps involving bifurcation in the ORR mechanism schematic; PDS for different CN*_x_* models within the independent site analysis; free energy for surface Pourbaix analysis of pyridinic and quaternary sites; detailed integrated ORR mechanisms for pyridinic and quaternary site models (PDF)
