# Supplementary material for: Oxygen Reduction Reaction on N‑Doped Graphene under Acidic Conditions: Integrating N‑Dopant Site Structure Changes with Competing Elementary Reaction Steps
Source: J Phys Chem C Nanomater Interfaces. 2026 Sep 23;130(40):13720–33. doi: 10.1021/acs.jpcc.6c04248 (PMC13656774; doi:10.1021/acs.jpcc.6c04248)
Supplement: Supplementary file 1 [file jp6c04248_si_001.pdf]

Supporting information

Oxygen reduction reaction on N-doped graphene under acidic conditions: Integrating N-dopant site structure changes with competing elementary reaction steps

Spand Mehta,† Aishwarya Rao,† and Aravind Asthagiri*

William G. Lowrie Department of Chemical and Biomolecular Engineering,

The Ohio State University, 151 W. Woodruff Avenue, Columbus, OH 43210, USA

† Spand Mehta and Aishwarya Rao contributed equally to this work.

*Corresponding author email: asthagiri.1@osu.edu

Section S1: **Site minimization procedure**

For each site model for CN_x shown in Fig. 1 in the manuscript, the adsorption of ORR intermediates on different sites adjacent to the N-dopant were examined and the configuration with minimum DFT energy is chosen for the evaluation of free energy for the intermediate. Below, the procedure for adsorption of O*, OH* and OOH* is shown for ZQ as an illustration.

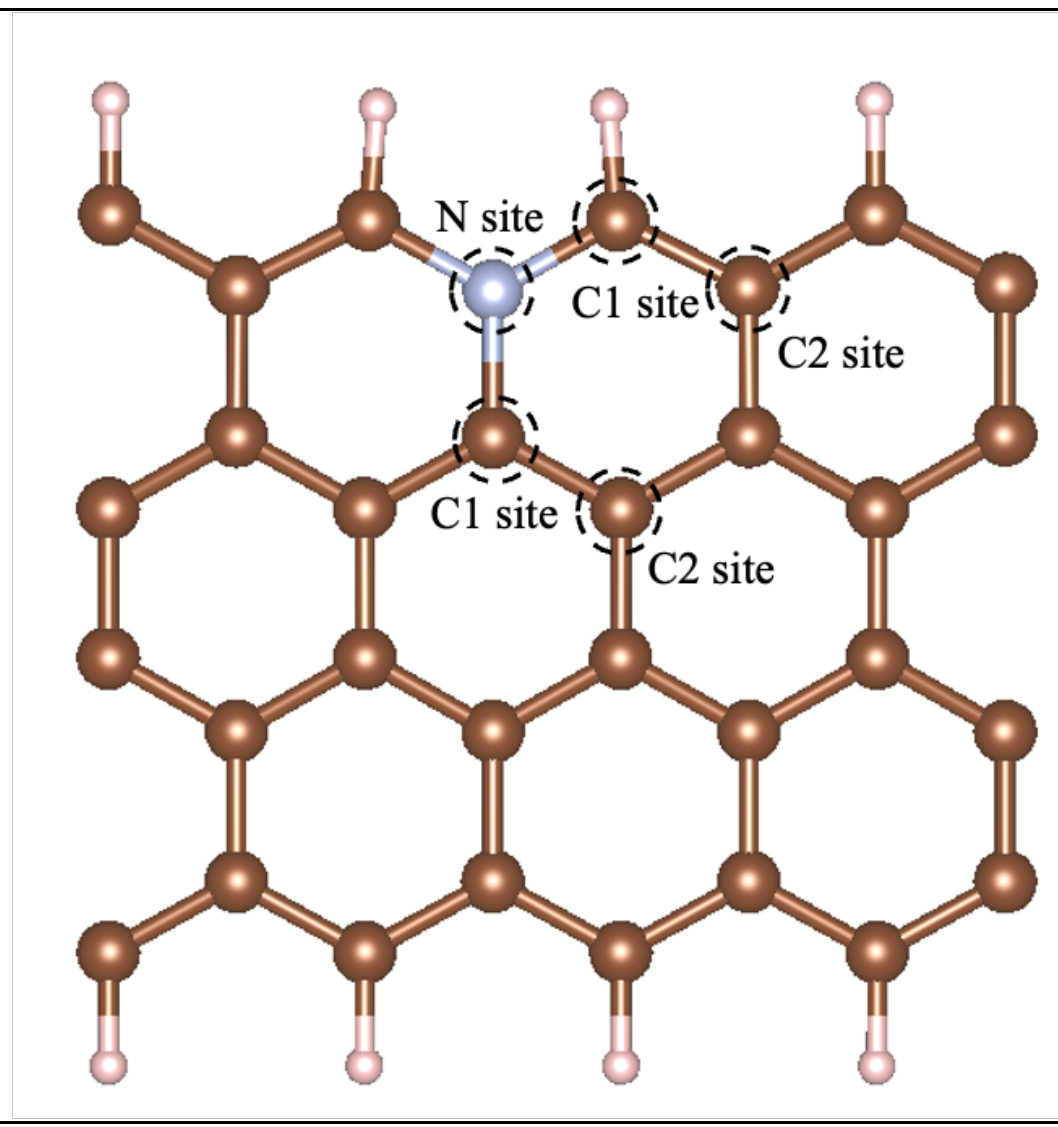

Figure S1. Different neighboring sites explored for ZQ CN_x site

a. O* adsorption

Adsorption of O directly on top of the N dopant along with adsorption on carbon sites near the nitrogen site (C1/C2) are considered. C1 is the carbon atom adjacent to nitrogen atom and C2 is second nearest carbon atom to nitrogen atom (see Fig. S1). The initial O configuration involves adsorbing O at 1.5 Å from the surface. The initial and final states along with the DFT energy is shown for different possible configurations in Table S1. The most negative DFT energy is chosen as the most stable/preferred configuration. We then perform VASPsol calculation for this most stable site and then perform free energy calculations.

Table S1: POSCAR and CONTCAR files for O* adsorption on ZQ for different sites

Initial configuration	Final configuration	Initial configuration	Final configuration
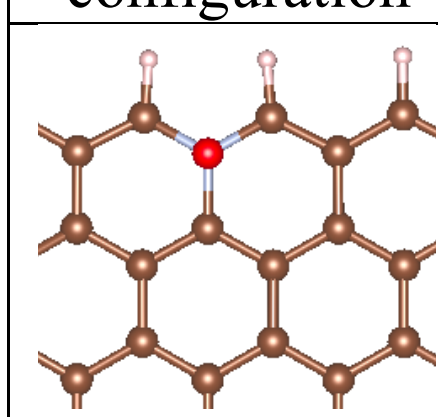	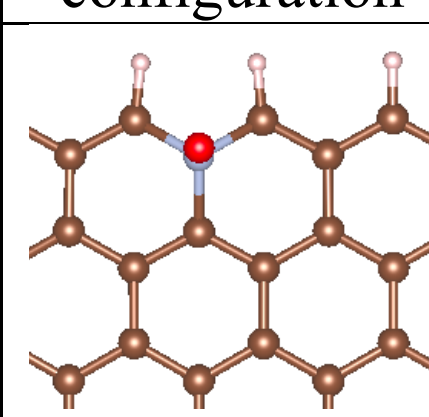	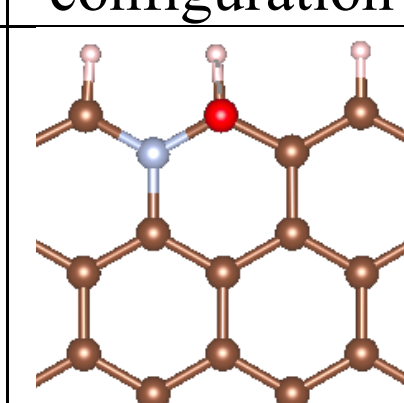	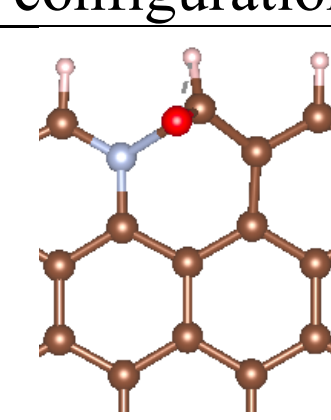
DFT Energy = -324.64 eV		DFT Energy = -326.80 eV	
Site 1		Site 2	

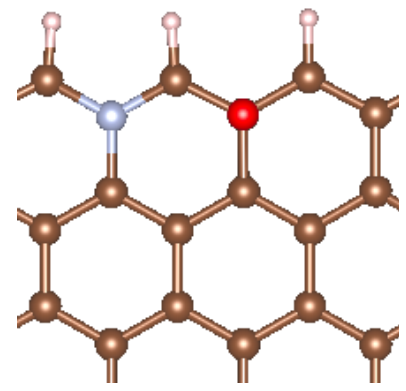	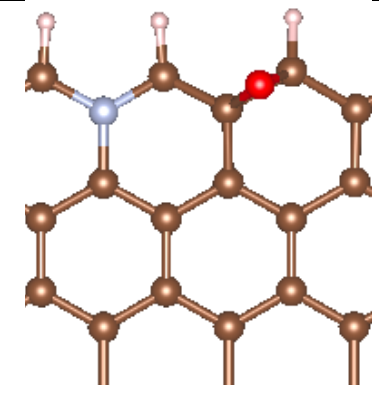	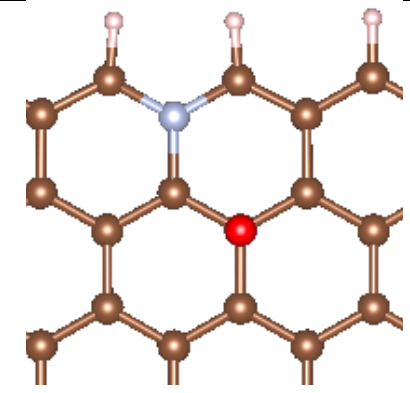	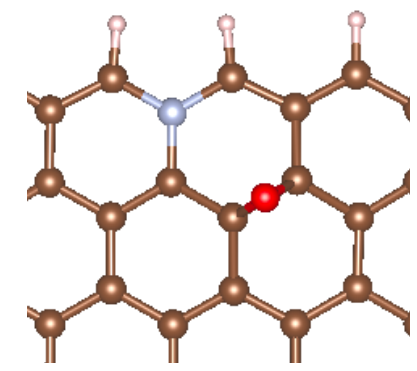
**DFT Energy = -327.07 eV**		DFT Energy = -326.44 eV	
**Site 3**		Site 4	

b. OH* adsorption

Similarly, OH is adsorbed on different sites as done for O adsorption. OH is initially assumed to be linear and it is adsorbed as atop configuration on top of a site with O at distance of 1.5 Å. The most stable configuration is highlighted and then VASPsol and free energy calculations carried.

Table S2: POSCAR and CONTCAR files for OH* adsorption on ZQ for different sites

Initial configuration	Final configuration	Initial configuration	Final configuration
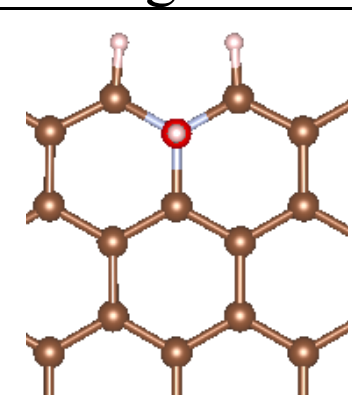	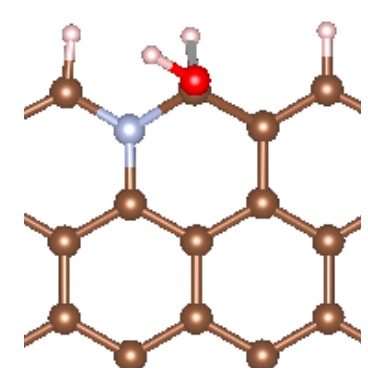	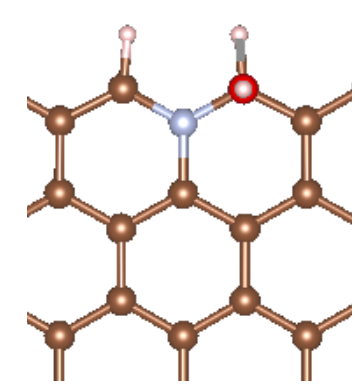	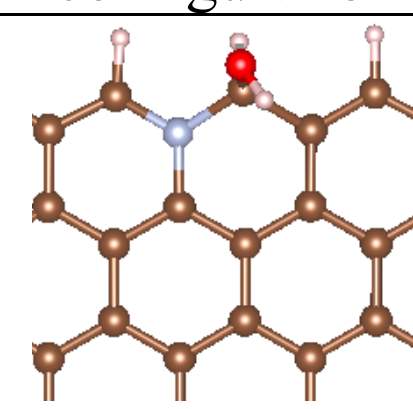
DFT Energy = -332.38 eV		**DFT Energy = -332.51 eV**	
Site 1		**Site 2**	
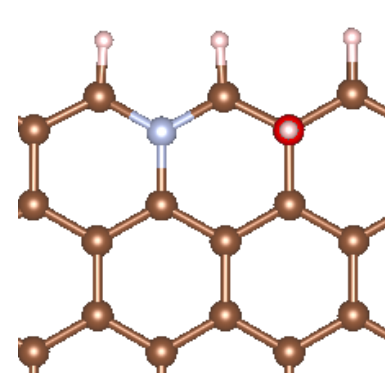	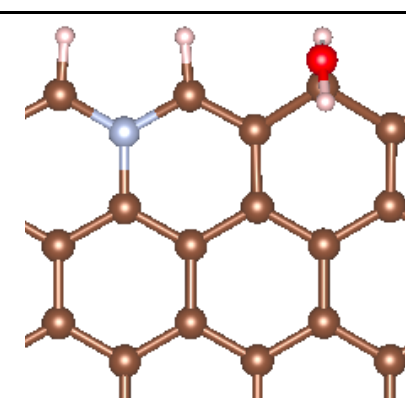	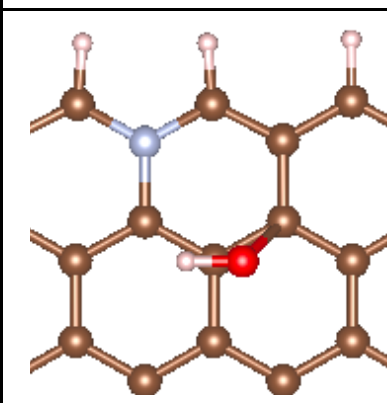	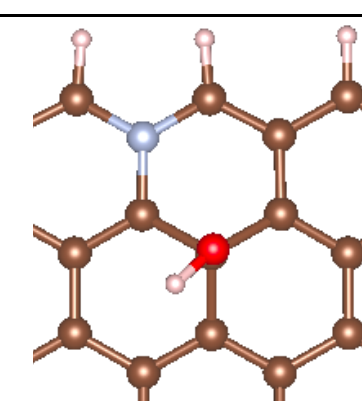
DFT Energy = -332.19 eV		DFT Energy = -330.89 eV	
Site 3		Site 4	

c. OOH* adsorption

OOH molecule being non-linear, we first carry out DFT energy optimization of OOH molecule. The relaxed configuration is then adsorbed on different configurations at a distance of 1.5 Å from the surface similar to procedure described above.

Table S3: POSCAR and CONTCAR files for OOH* adsorption on ZQ for different sites

Initial config	Final config	Initial config	Final config	Initial config	Final config

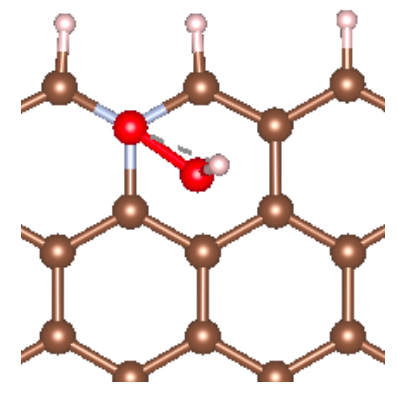	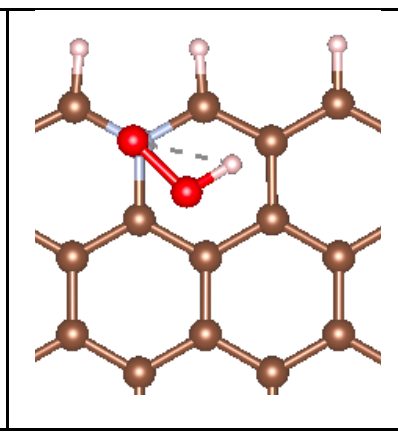	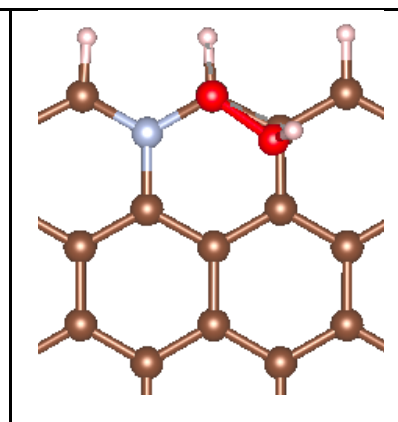	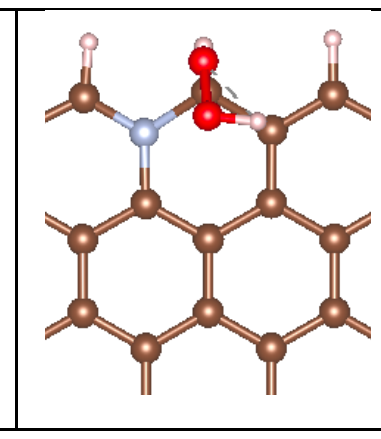	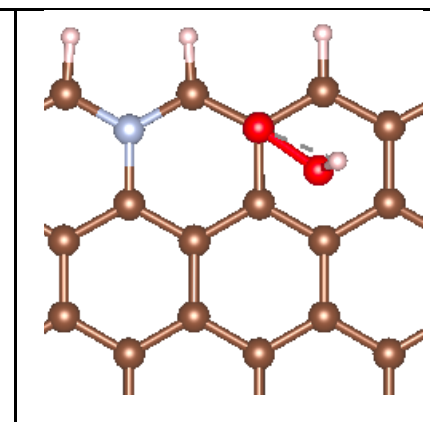	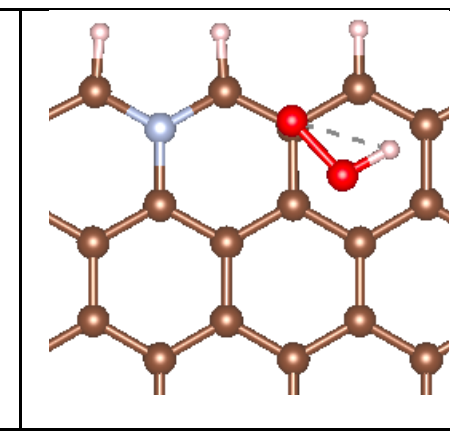
DFT Energy = -335.67 eV		**DFT Energy = -336.70 eV**		DFT Energy = -335.64 eV	
Site 1		**Site 2**		Site 3	
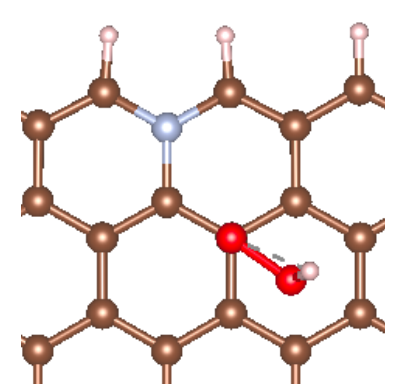	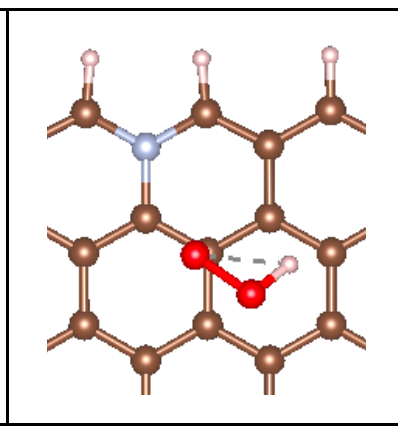				
DFT Energy = -335.65 eV					
Site 4					

d. O*+OH* adsorption

In this case, we first consider the DFT optimized structure of O*. We then adsorb OH* on different sites (on and nearby N atom) and then most stable configuration is chosen. To confirm, that this procedure gives the stable site, we have also considered all possible combinations of O* and OH* on different sites (atop configuration), which yields the same structure. The latter part is only done for selected CN_x models since it involves several combinations, making it computationally expensive.

Table S4: POSCAR and CONTCAR files for O*+OH* adsorption on ZQ for different sites

Initial config	Final config	Initial config	Final config	Initial config	Final config
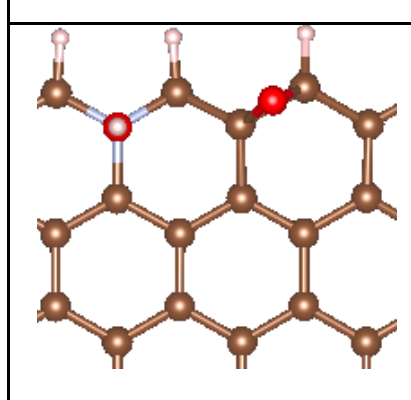	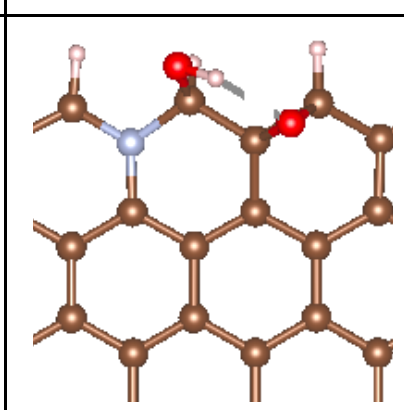	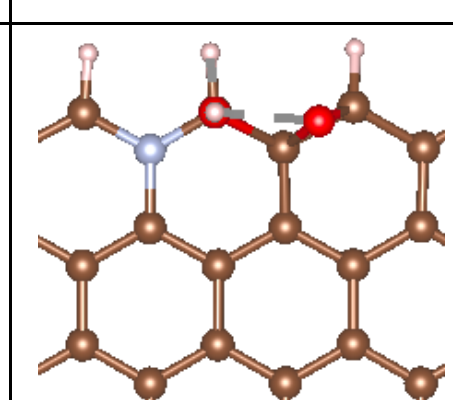	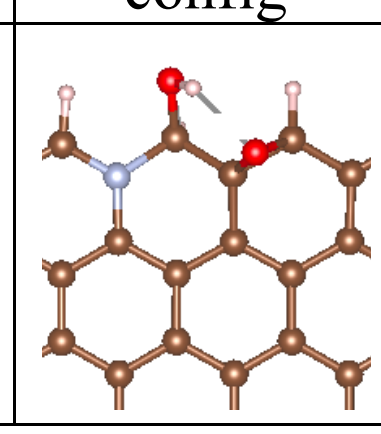	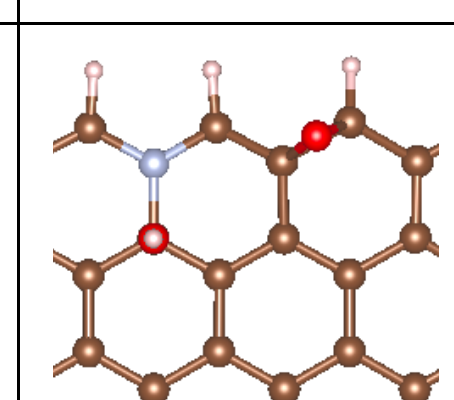	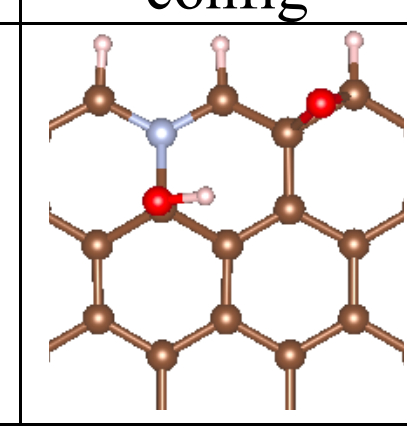
DFT Energy = -337.75 eV		**DFT Energy = -338.01 eV**		DFT Energy = -336.35 eV	
Site 1		**Site 2**		Site 3	
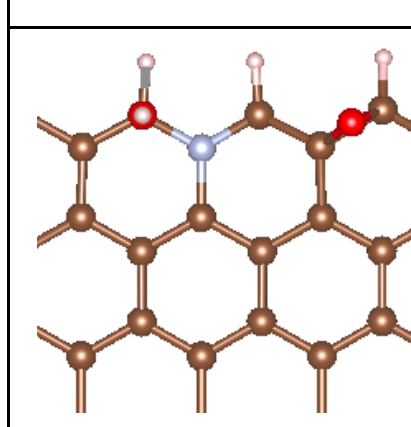	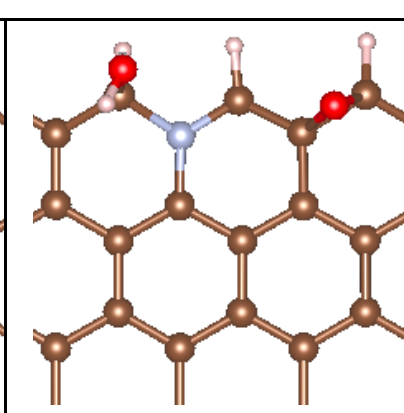	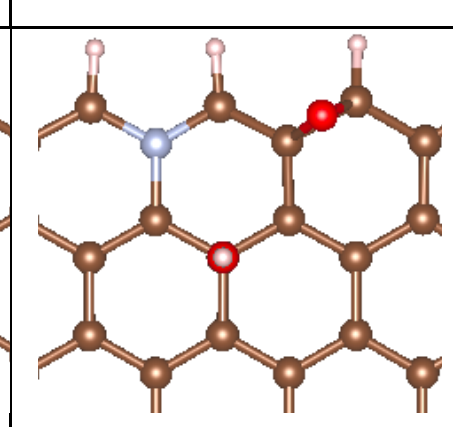	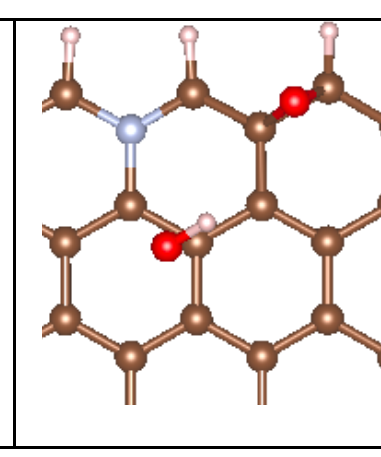	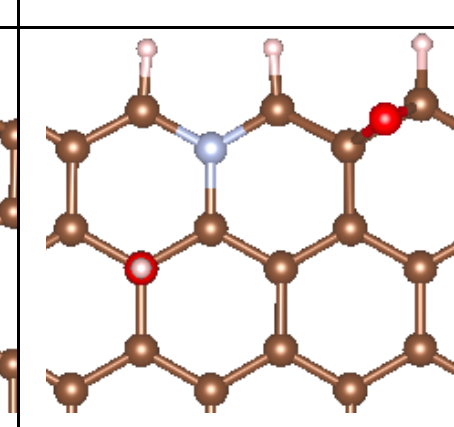	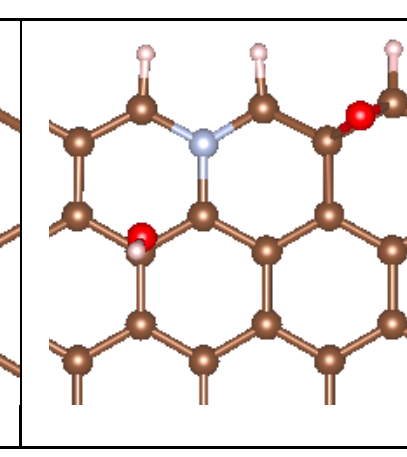
DFT Energy = -337.29 eV		DFT Energy = -335.40 eV		DFT Energy = -335.37 eV	
Site 4		Site 5		Site 6	

Section S2: **Evaluation of competition between elementary steps involving bifurcation in the ORR mechanism schematic**

Within Scheme 1 the O*+OH* species can follow two pathways and Table S5 shows the ΔG for these alternate steps. For all CN_x models, we find that ΔG(O*+OH*→O*) is more negative than ΔG(O*+OH*→OH*+OH*). Thus, ΔG(O*+OH*→OH*+OH*) is not favorable for any CN_x model and thus not considered in the independent site analysis.

Table S5: Competition of pathways, O*+OH*→O* and O*+OH*→OH*+OH*. Free energy is reported at 1 V-RHE and in units of eV.

Model Classification	Models	ΔG(O*+OH*→O*)	ΔG (O*+OH*→OH*+OH*)
AP Models	AP	-1.39	-0.93
	AP-H	-0.38	0.17
	AP-O	-0.62	0.23
ZP Models	ZP	0.26	1.31
	ZP-H	-0.55	-0.12
	ZP-O	0.12	1.17
Pyr Models	Pyr	0.03	1.10
	Pyr - H	-0.62	-0.62
	PyrO	-0.40	0.05
Quaternary Models	ZQ	0.29	0.80
	AQ	-0.14	0.82
	BQ	-0.61	1.00

Section S3: The PDS for different CN_x models within the independent site analysis

Table S6 shows the elementary step and thermodynamic limiting potential for all the CNx site models considered in the independent site analysis. The results for both the associative and dissociative mechanism branches of the ORR in Scheme 1 are shown.

Table S6: The PDS for associative and dissociative mechanism along with thermodynamic limiting potential in V-RHE is reported in brackets. The CN_x site models are classified into armchair pyridinic (AP), zigzag pyridinic (ZP), pyrrolic (Pyr) and quaternary models.

Model Classification	Models	Associative mechanism	Dissociative mechanism
AP Models	AP	O_2 → OOH* (0.27)	O_2 → O*+OH* (0.27)
	AP-H	OH* → H_2O (0.63)	OH* → H_2O (0.63)
	AP-O	O_2 → OOH* (0.64)	O_2 → O*+OH* (0.90)
ZP Models	ZP	OH* → H_2O (0.52)	OH* → H_2O (0.52)
	ZP-H	O_2 → OOH* (0.40)	O* → OH* (0.72)
	ZP-O	OH* → H_2O (0.66)	OH* → H_2O (0.66)
Pyr Models	Pyr	OH* → H_2O (0.66)	OH* → H_2O (0.66)
	Pyr-H	O_2 → OOH* (0.34)	O* → OH* (0.61)
	PyrO	O_2 → OOH* (0.93)	OH* → H_2O (0.95)
Quaternary Models	ZQ	OH* → H_2O (0.57)	O* +OH* → O* (0.27)
	AQ	OH* → H_2O (0.87)	OH* → H_2O (0.87)
	BQ	O_2 → OOH* (0.77)	O_2 → O*+OH* (0.99)

Section S4: **Free energy for surface Pourbaix analysis of pyridinic and quaternary sites**

Table S7 contains the free energy to form various surface species shown in Table 1. All energies are referenced to the bare CN_x site model. This data is used to generate the surface Pourbaix diagrams (Fig. 4-6) shown in the paper.

Table S7: The values of a and b corresponding the respective structures for pyridinic and quaternary structures along with respective free energy of reaction (eV) at 1.23-V RHE. For quaternary structures, only X/O* and X/OH* were stable and thus free energies of other structures are not reported.

a	b	Y	ZP	AP	Pyr	ZQ	AQ	BQ
0	1	X-H	0.01	0.92	-0.62	NA	NA	NA
1	-2	X-O	-0.84	-0.31	-1.22	NA	NA	NA
1	-1	X-OH	-0.17	1.15	-0.77	NA	NA	NA
1	-2	X/O*	-0.76	0.10	-1.14	-0.67	-0.66	-0.14
1	-1	X/OH*	-0.53	0.38	-0.57	-0.66	-0.30	-0.13
2	-4	X-O/O*	-0.99	-0.37	-1.41	NA	NA	NA
2	-3	X-O/OH*	-1.41	-0.45	-1.50	NA	NA	NA
2	-3	X-OH/O*	-0.62	0.15	-1.08	NA	NA	NA
2	-2	X-OH/OH*	0.38	-0.21	-0.30	NA	NA	NA
1	-1	X-H/O*	-0.37	0.30	-0.82	NA	NA	NA
1	0	X-H/OH*	0.14	0.32	-0.29	NA	NA	NA

Section S5: Integrated ORR mechanism for pyridinic models

Protonation/ORR steps for all the pyridinic models showing competing protonation and oxygen addition steps. The most preferred pathways are highlighted in red. Note Fig. 7 (ZP) is reproduced below for convenience. The dashed arrow(s) indicate an alternative pathway that is within 0.05 eV of the most preferred pathway.

Pyridinic models

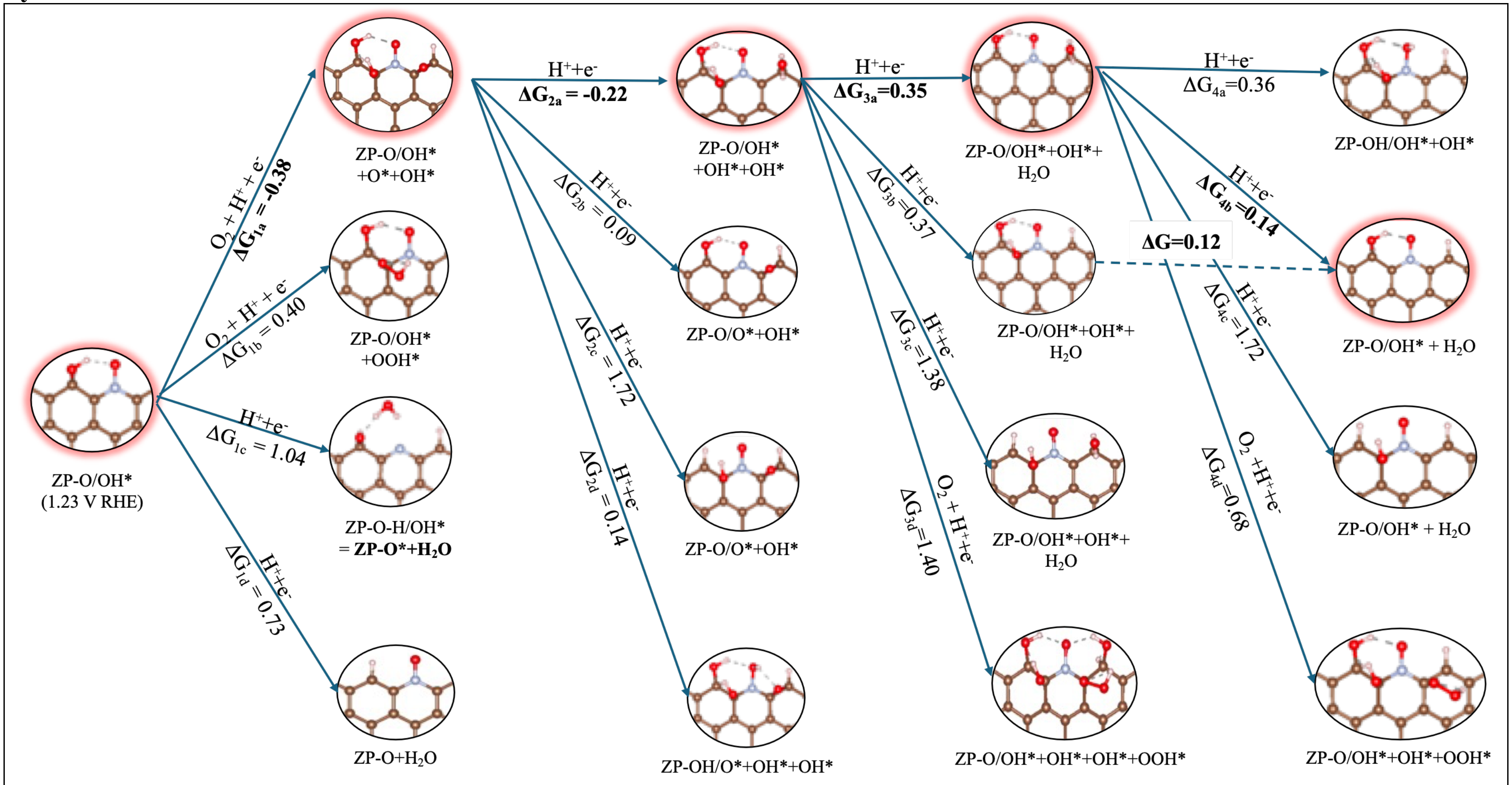

ZP

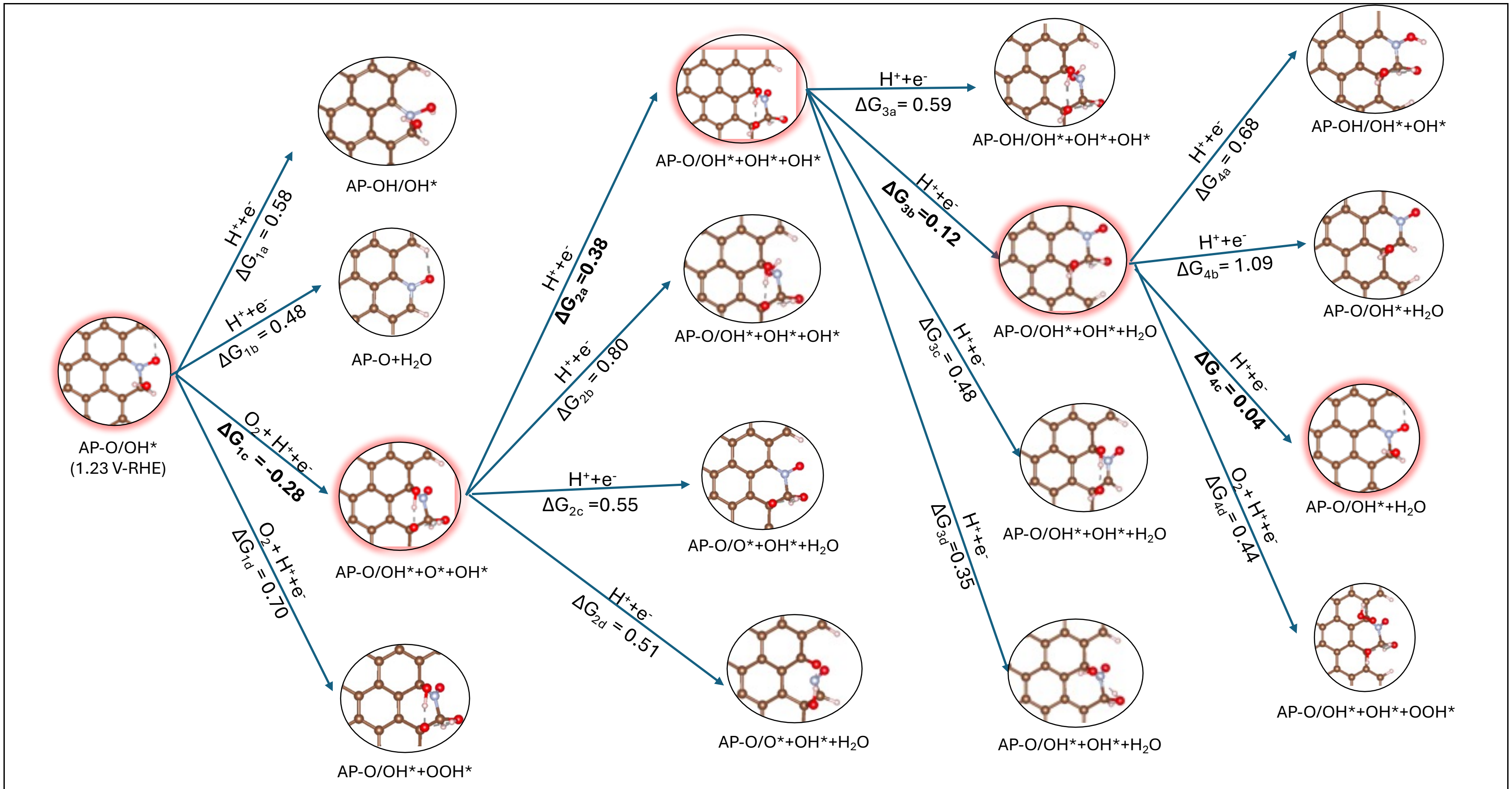
AP-O/OH*
(1.23 V-RHE)
H+e-
ΔG1a = 0.58
AP-OH/OH*
H+e-
ΔG1b = 0.48
AP-O+H2O
O2+H++e-
ΔG1c = -0.28
AP-O/OH*+O*+OH*
O2+H++e-
ΔG1d = 0.70
AP-O/OH*+OOH*
H+e-
ΔG2a =0.38
AP-O/OH*+OH*+OH*
H+e-
ΔG2b = 0.80
AP-O/OH*+OH*+OH*
H+e-
ΔG2c =0.55
AP-O/O*+OH*+H2O
H+e-
ΔG2d = 0.51
AP-O/O*+OH*+H2O
H+e-
ΔG3a= 0.59
AP-OH/OH*+OH*+OH*
H+e-
ΔG3b =0.12
AP-O/OH*+OH*+H2O
H+e-
ΔG3c = 0.48
AP-O/OH*+OH*+H2O
H+e-
ΔG3d=0.35
AP-O/OH*+OH*+H2O
H+e-
ΔG4a = 0.68
AP-OH/OH*+OH*
H+e-
ΔG4b= 1.09
AP-O/OH*+H2O
H+e-
ΔG4c = 0.04
AP-O/OH*+H2O
O2+H++e-
ΔG4d = 0.44
AP-O/OH*+OH*+OOH*

AP

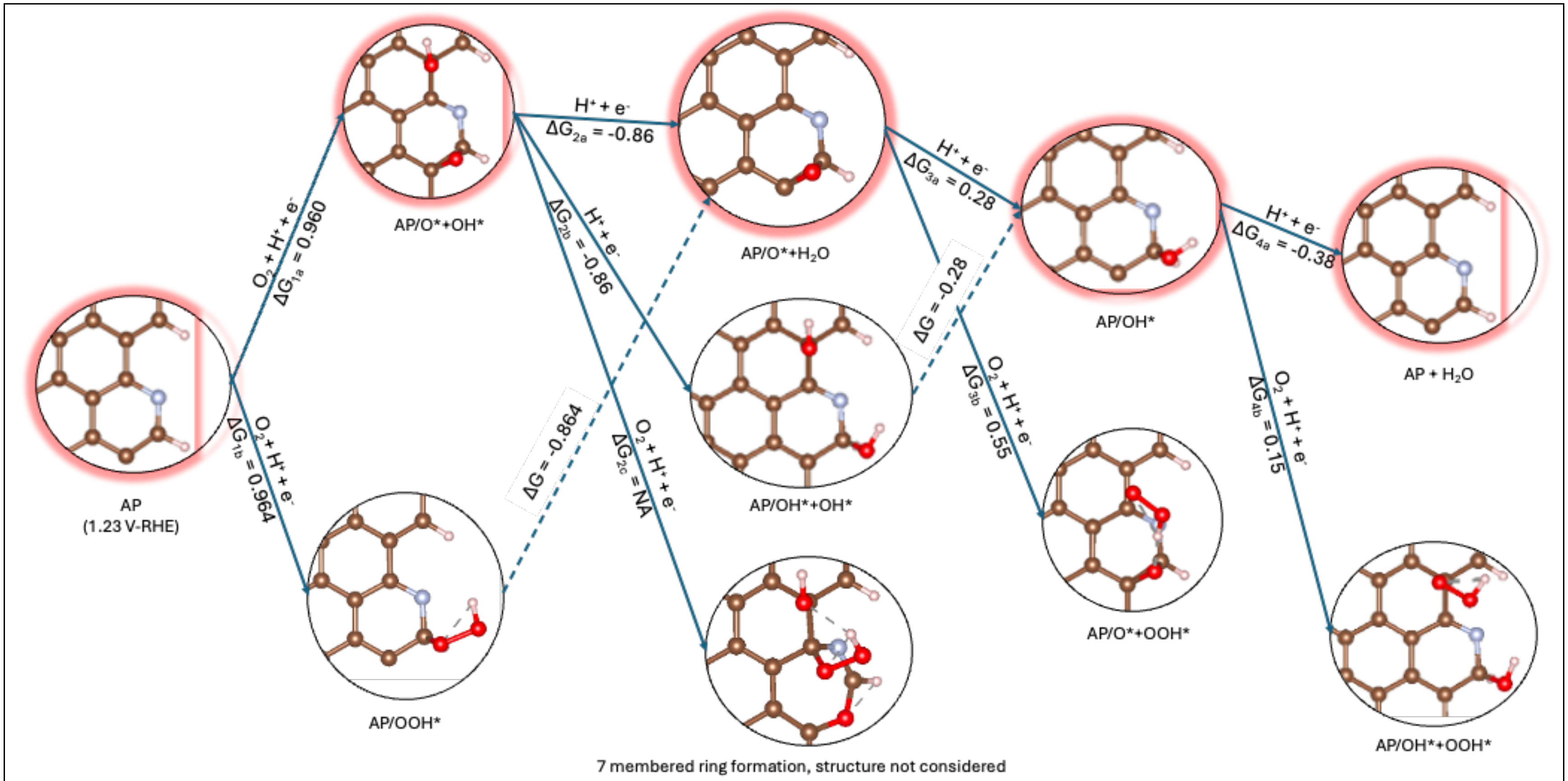

AP (when oxidization at 1.23 V-RHE is kinetically hindered)

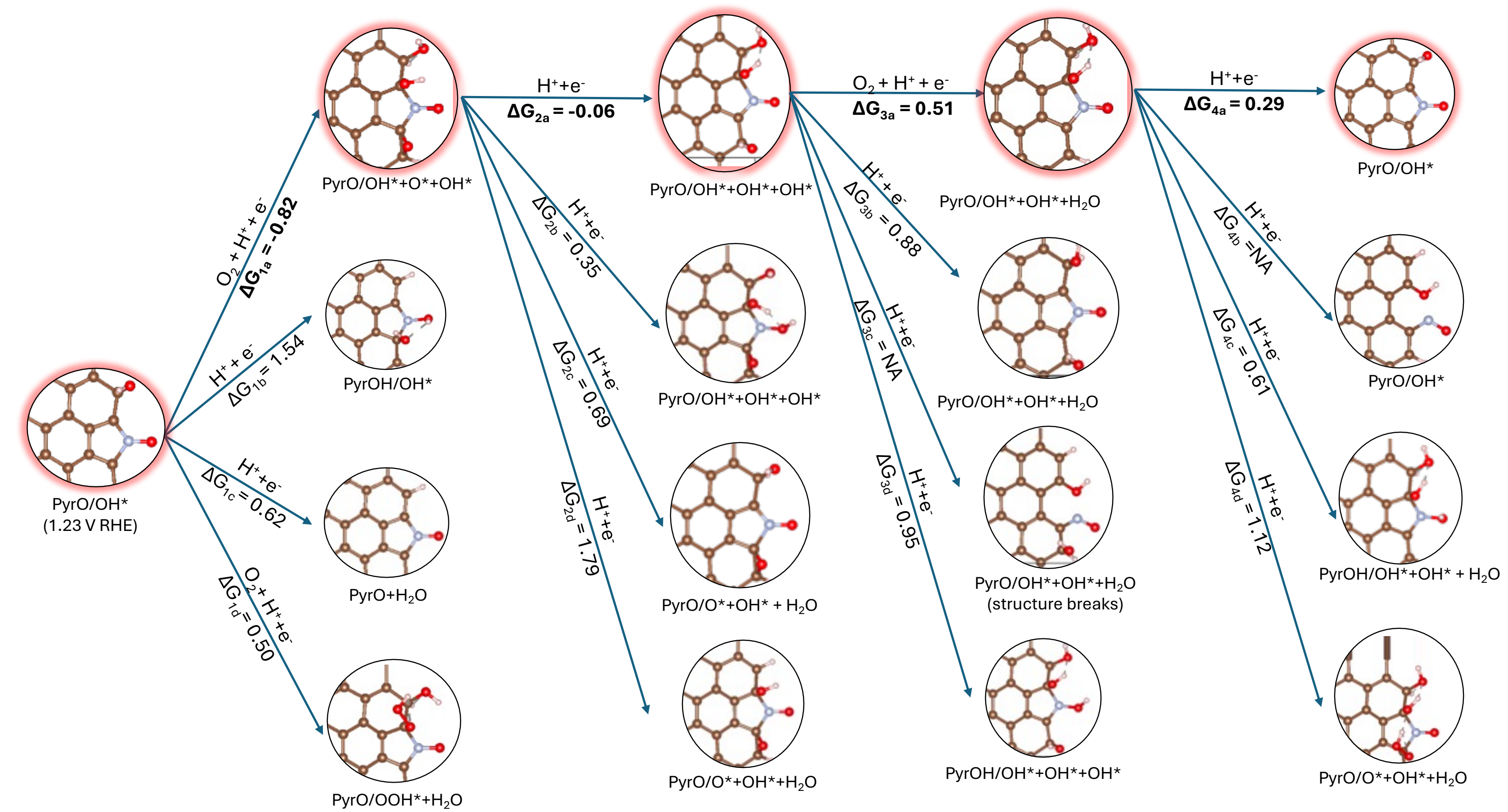

PyrO/OH*
(1.23 V RHE)
O2 + H+ + e-
ΔG1a = -0.82
H+ + e-
ΔG1b = 1.54
H+ + e-
ΔG1c = 0.62
O2 + H+ + e-
ΔG1d = 0.50
PyrO/OH*+O*+OH*
PyrOH/OH*
PyrO+H2O
PyrO/OOH*+H2O
H+ + e-
ΔG2a = -0.06
H+ + e-
ΔG2b = 0.35
H+ + e-
ΔG2c = 0.69
H+ + e-
ΔG2d = 1.79
PyrO/OH*+OH*+OH*
PyrO/OH*+OH*+OH*
PyrO/O*+OH* + H2O
PyrO/O*+OH*+H2O
O2 + H+ + e-
ΔG3a = 0.51
H+ + e-
ΔG3b = 0.88
H+ + e-
ΔG3c = NA
H+ + e-
ΔG3d = 0.95
PyrO/OH*+OH*+H2O
PyrO/OH*+OH*+H2O
PyrO/OH*+OH*+H2O
(structure breaks)
PyrOH/OH*+OH*+OH*
H+ + e-
ΔG4a = 0.29
H+ + e-
ΔG4b = NA
H+ + e-
ΔG4c = 0.61
H+ + e-
ΔG4d = 1.12
PyrO/OH*
PyrO/OH*
PyrOH/OH*+OH* + H2O
PyrO/O*+OH*+H2O

Pyr

Section S6: Integrated ORR mechanism for quaternary models

Protonation/ORR steps for all the quaternary models showing competing protonation and oxygen addition steps. The most preferred pathways are highlighted in red. Note for quaternary models we do not find any alternative steps within 0.05 eV of the most preferred pathways.

Quaternary models

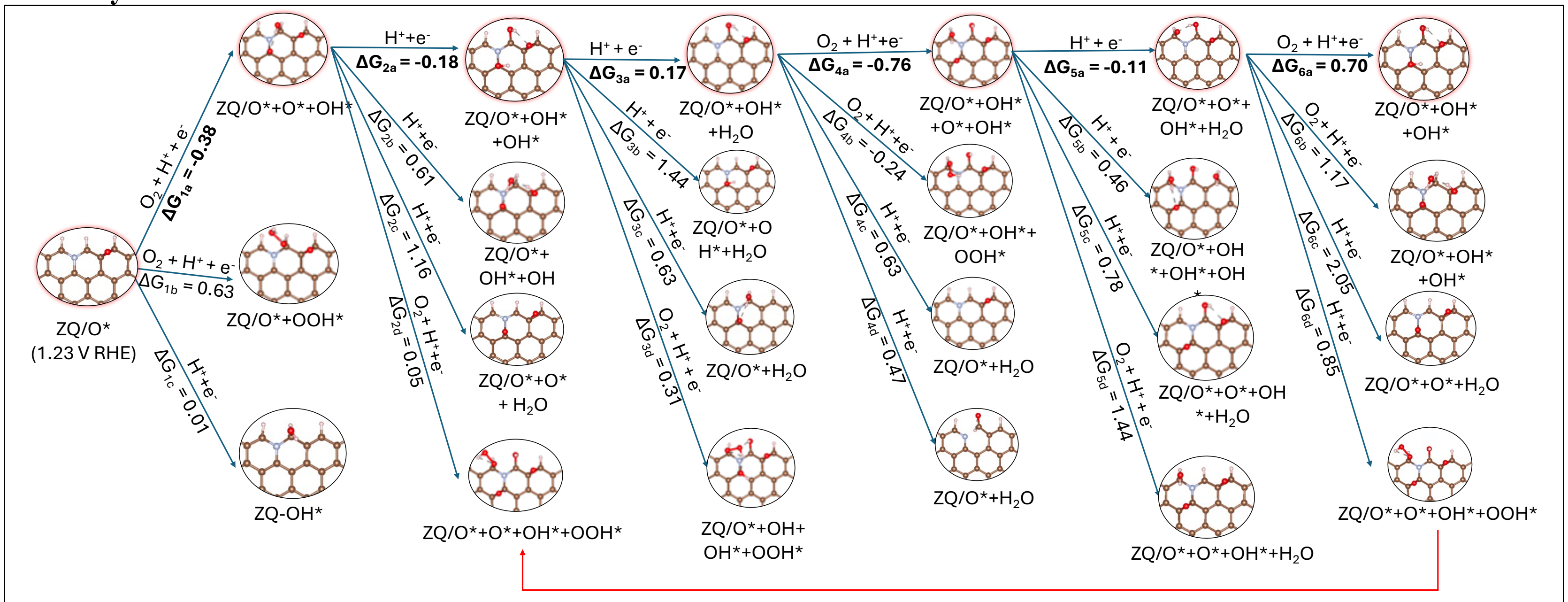

ZQ

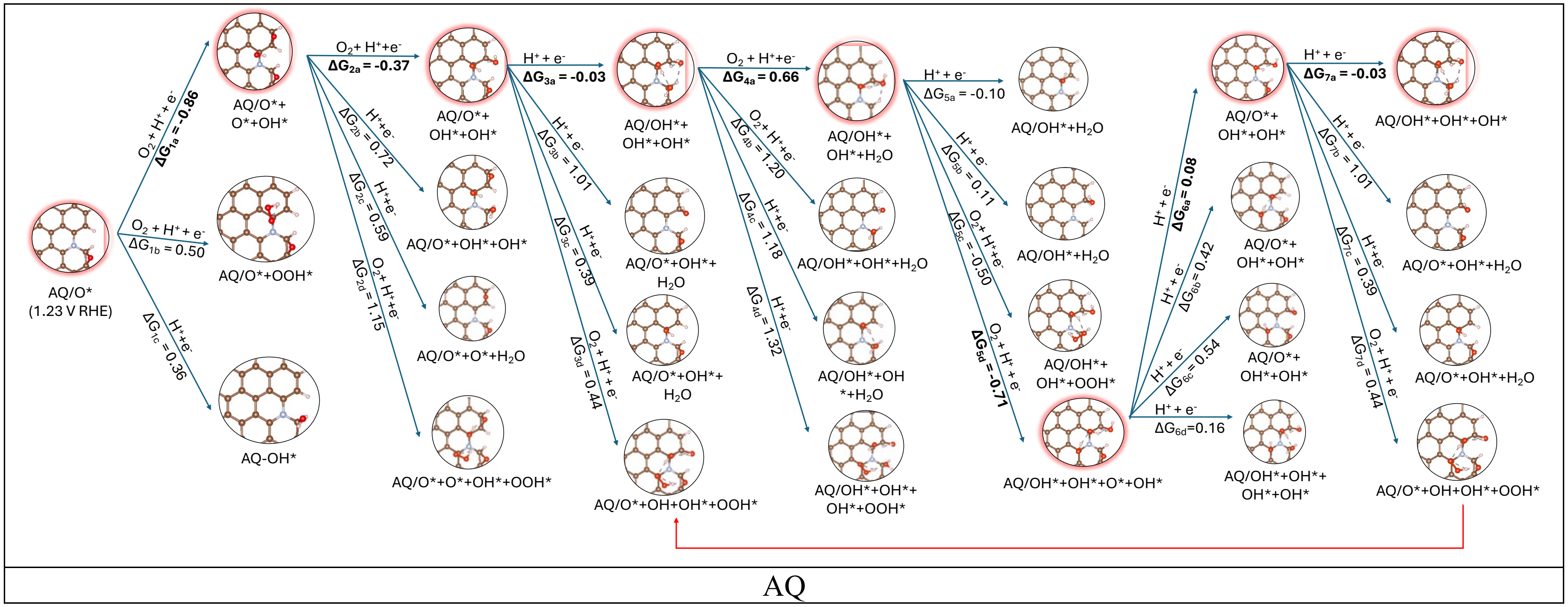
AQ/O*
(1.23 V RHE)
$O_2 + H^+ + e^-$ ΔG_{1a} = -0.86
AQ/O*+ O*+OH*
$O_2 + H^+ + e^-$ ΔG_{1b} = 0.50
AQ/O*+OOH*
$H^+ + e^-$ ΔG_{1c} = 0.36
AQ-OH*
$O_2 + H^+ + e^-$ ΔG_{2a} = -0.37
AQ/O*+ OH*+OH*
$H^+ + e^-$ ΔG_{2b} = 0.72
AQ/O*+OH*+OH*
$H^+ + e^-$ ΔG_{2c} = 0.59
AQ/O*+O*+H_2O
$O_2 + H^+ + e^-$ ΔG_{2d} = 1.15
AQ/O*+O*+OH*+OOH*
$H^+ + e^-$ ΔG_{3a} = -0.03
AQ/OH*+ OH*+OH*
$H^+ + e^-$ ΔG_{3b} = 1.01
AQ/O*+OH*+ H_2O
$H^+ + e^-$ ΔG_{3c} = 0.39
AQ/O*+OH*+ H_2O
$O_2 + H^+ + e^-$ ΔG_{3d} = 0.44
AQ/O*+OH+OH*+OOH*
$O_2 + H^+ + e^-$ ΔG_{4a} = 0.66
AQ/OH*+ OH*+H_2O
$O_2 + H^+ + e^-$ ΔG_{4b} = 1.20
AQ/OH*+OH*+H_2O
$H^+ + e^-$ ΔG_{4c} = 1.18
AQ/OH*+OH *+H_2O
$H^+ + e^-$ ΔG_{4d} = 1.32
AQ/OH*+OH*+ OH*+OOH*
$H^+ + e^-$ ΔG_{5a} = -0.10
AQ/OH*+H_2O
$H^+ + e^-$ ΔG_{5b} = 0.11
AQ/OH*+H_2O
$O_2 + H^+ + e^-$ ΔG_{5c} = -0.50
AQ/OH*+ OH*+OOH*
$O_2 + H^+ + e^-$ ΔG_{5d} = -0.71
AQ/OH*+OH*+O*+OH*
$H^+ + e^-$ ΔG_{6a} = 0.08
AQ/O*+ OH*+OH*
$H^+ + e^-$ ΔG_{6b} = 0.42
AQ/O*+ OH*+OH*
$H^+ + e^-$ ΔG_{6c} = 0.54
AQ/O*+ OH*+OH*
$H^+ + e^-$ ΔG_{6d} = 0.16
AQ/OH*+OH*+ OH*+OH*
$H^+ + e^-$ ΔG_{7a} = -0.03
AQ/OH*+OH*+OH*
$H^+ + e^-$ ΔG_{7b} = 1.01
AQ/O*+OH*+H_2O
$H^+ + e^-$ ΔG_{7c} = 0.39
AQ/O*+OH*+H_2O
$O_2 + H^+ + e^-$ ΔG_{7d} = 0.44
AQ/O*+OH+OH*+OOH*
AQ

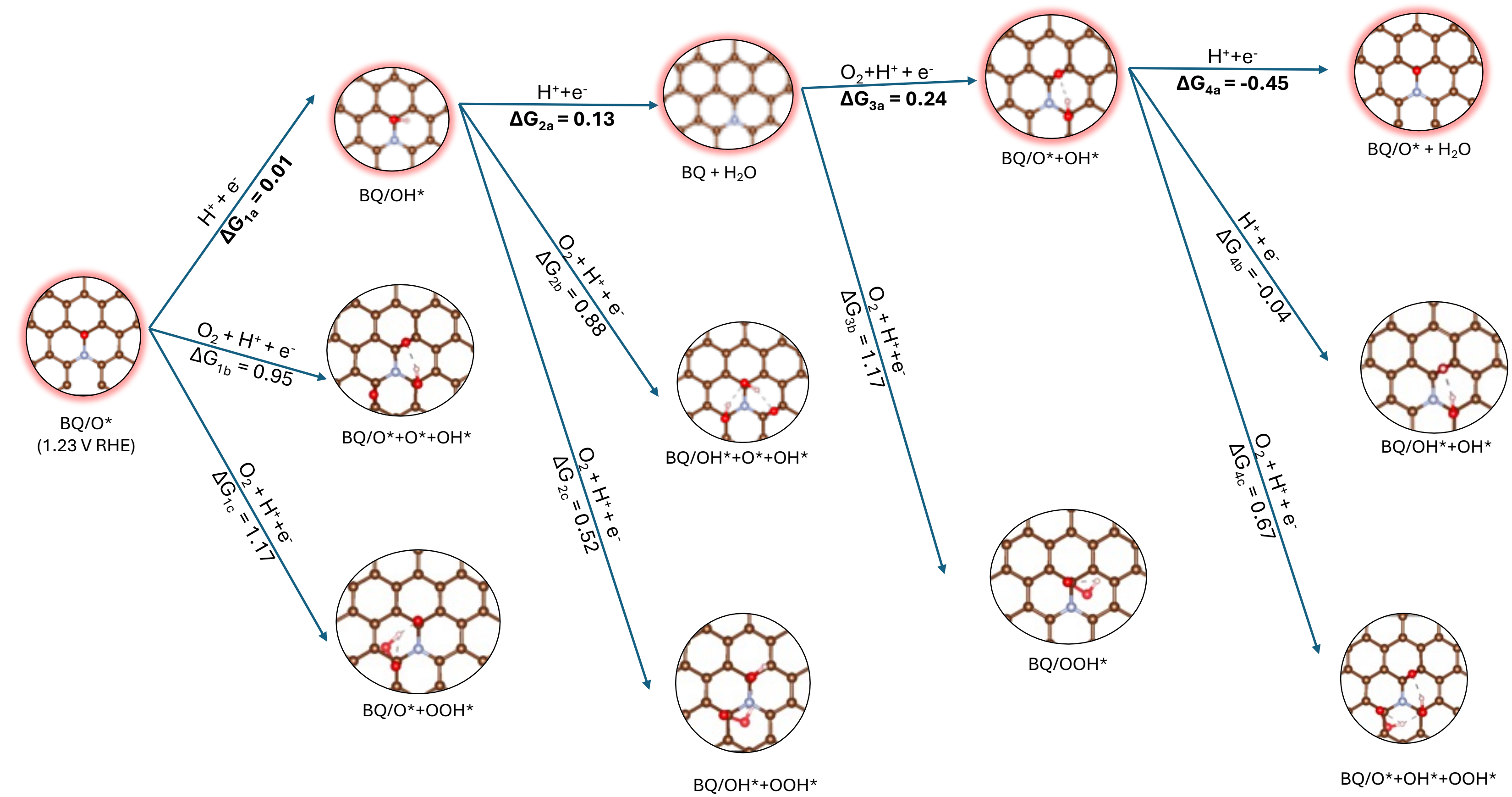
BQ/O*
(1.23 V RHE)
H⁺ + e⁻
ΔG1a = 0.01
BQ/OH*
O2 + H⁺ + e⁻
ΔG1b = 0.95
BQ/O*+O*+OH*
O2 + H⁺+e⁻
ΔG1c = 1.17
BQ/O*+OOH*
H⁺+e⁻
ΔG2a = 0.13
BQ + H2O
O2 + H⁺ + e⁻
ΔG2b = 0.88
BQ/OH*+O*+OH*
O2 + H⁺ + e⁻
ΔG2c = 0.52
BQ/OH*+OOH*
O2+H⁺ + e⁻
ΔG3a = 0.24
BQ/O*+OH*
O2 + H⁺+e⁻
ΔG3b = 1.17
BQ/OOH*
H⁺+e⁻
ΔG4a = -0.45
BQ/O* + H2O
H⁺ + e⁻
ΔG4b = -0.04
BQ/OH*+OH*
O2 + H⁺ + e⁻
ΔG4c = 0.67
BQ/O*+OH*+OOH*

BQ

Section S7: Consideration of additional adjacent sites for ORR intermediates

As noted in the manuscript, for ORR integrated mechanism we imposed a rule that additional O_2 reaction products (OOH* or (O* + OH*)) would be only examined on free carbon sites directly adjacent to the dopant N (labeled C1 sites). In this section, we explore free carbon sites located adjacent to the C1 sites (labeled C2 sites) for the ZP and BQ sites. As before we are performing an integrated mechanism study where multiple possible next steps in the pathway are evaluated, with the only difference being that the allowed configurational search space has been expanded to include C2 sites for both (OH*+O*) and OOH* formation. We have chosen ZP and BQ as these are active sites within the integrated framework and a good test case to see if the original rule limiting the configurational search space is reasonable.

We examine every step of the overall reaction cycle of ZP and BQ shown in Fig. 8a and 9c, respectively, and evaluate energies for the obtained minima from the additional sites of O*+OH*/OOH*. Details of this extended search are shown below, but overall, for both ZP and BQ we do not find a preference for any of these additional configurations. Based on these results we assume that the general rule restricting the additional O_2-related species to open C1 sites is a reasonable approach.

ZP additional search configurations

Below we present the extended search for each step in the ORR mechanism. Table S8a-h shows ΔE (eV) values of these structures relative to the most stable site. The free energy at 1.23 V-RHE is also reported for the most stable site and compared with the corresponding step in the overall cycle in the table caption.

<u>Alternatives for the first step</u>

From Figure 7 and 8a, we can see the steps in ZP integrated ORR mechanism and the corresponding overall cycle, respectively. From the overall cycle, the preferred first step is ZP-O/OH* → ZP-O/OH*+O*+OH* (ΔG = -0.38 eV at 1.23 V-RHE). Thus, the first step favors adsorption of O*+OH* on the C1 sites. We now explore additional O*+OH* (Table S8a) and OOH* (Table S8b) sites to check if this remains true.

Table S8a: Relative energies of all O*+OH* configurations explored for the ZP-O/OH*→ ZP-O/OH*+O*+OH* step. The most stable site has ΔG = -0.38 eV at 1.23 V-RHE and is the same structure that was identified as the most favored in the existing integrated cycle.

Site 1	Site 2	Site 3	Site 4	Site 5
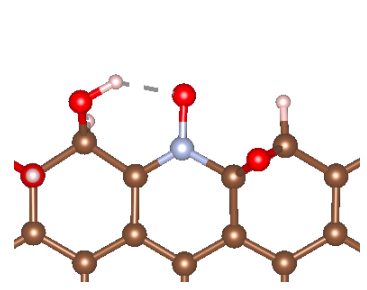	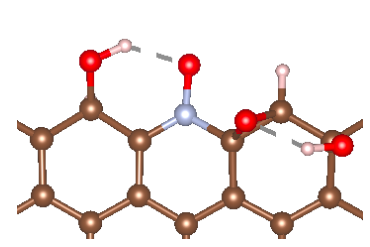	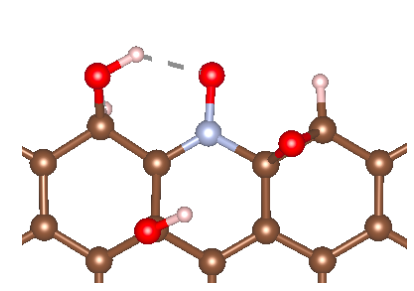	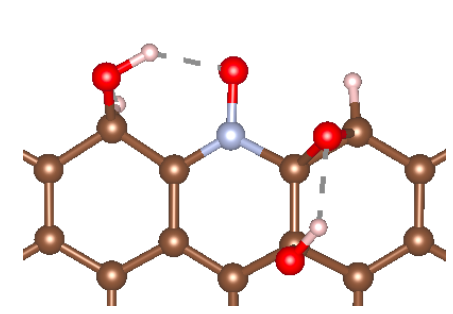	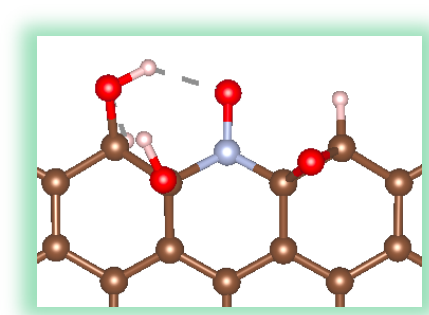
ΔE = 0.40	ΔE = 0.81	ΔE = 0.84	ΔE = 0.72	**ΔE = 0.00**

As shown above, the most stable O*+OH* configuration identified from the extended site search corresponds to the structure selected in the integrated ORR mechanism. We next examine whether a similar conclusion holds for OOH* adsorption.

Table S8b: Relative energies of all OOH* configurations explored for the ZP-O/OH* → ZP-O/OH*+OOH* step. The most stable site has ΔG = 0.40 eV at 1.23 V-RHE and is the same structure that was identified in the earlier search.

Site 1	Site 2	Site 3	Site 4	Site 5
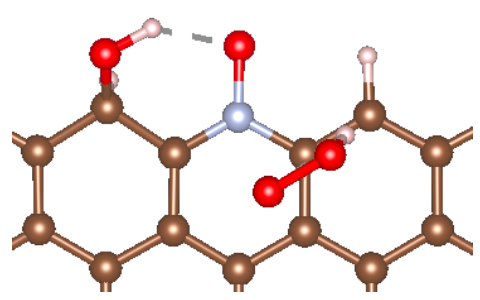	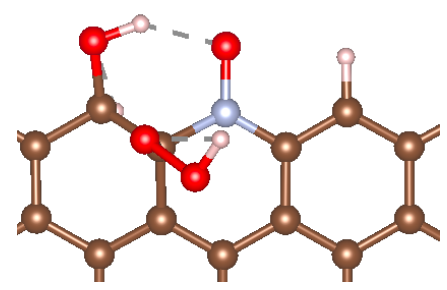	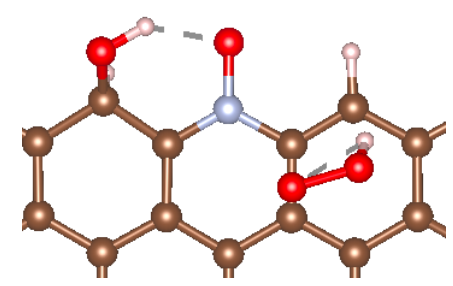	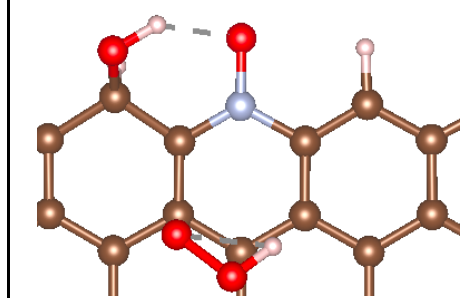	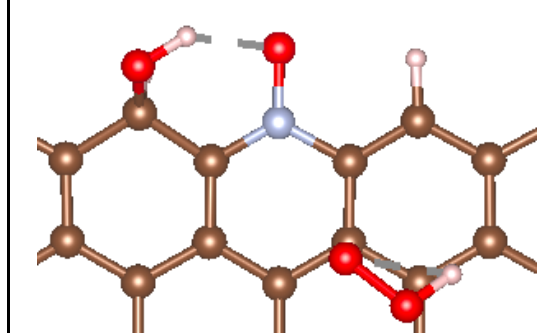
ΔE = 0.32	**ΔE = 0**	ΔE = 0.32	ΔE = 0.4	ΔE = 0.38

The most stable OOH* configuration identified from the extended site search is Site 2, which matches the original configuration in Fig. 7 for OOH* formation. Therefore, the extended search does not identify a new favorable OOH* configuration.

<u>Alternatives for the second step</u>

For the second step, most preferred step was a protonation of the existing O* to OH* given by ZP-O/OH*+O*+OH* → ZP-O/OH*+OH*+OH* (ΔG = -0.22 eV at 1.23 V-RHE). Based on our existing rule we did not explore any reaction products for O_2 on the surface since there is already existing O*/OH* on all the C1 sites. In Table S8c and S8d below we explore additional sites for the second step where O_2 reacts to form an additional OOH* and (O*+OH*), respectively.

Table S8c: Relative energies for all O*+OH* configurations explored for the ZP-O/OH*+O*+OH* → ZP-O/OH*+O*+OH*+O*+OH* step. The most stable site has ΔG = 0.36 eV at 1.23 V-RHE and this value is higher in free energy than the preferred step in the current mechanism.

Site 1	Site 2	Site 3	Site 4	Site 5	Site 6
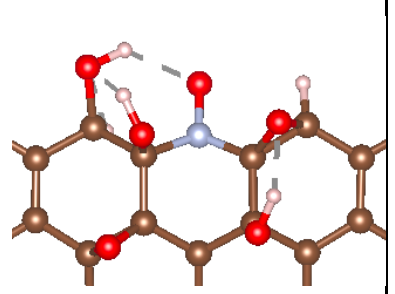	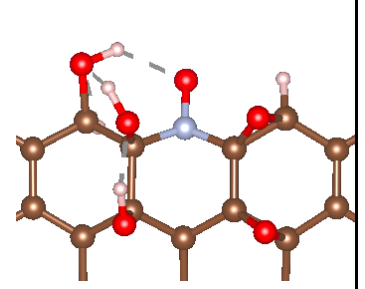	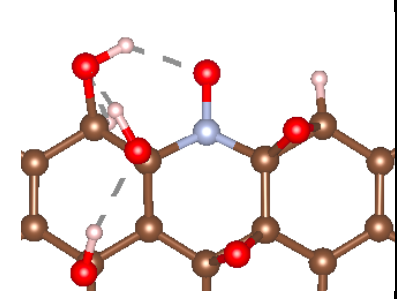	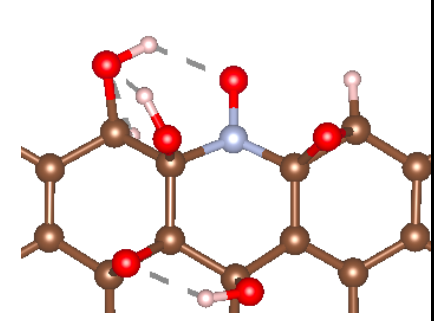	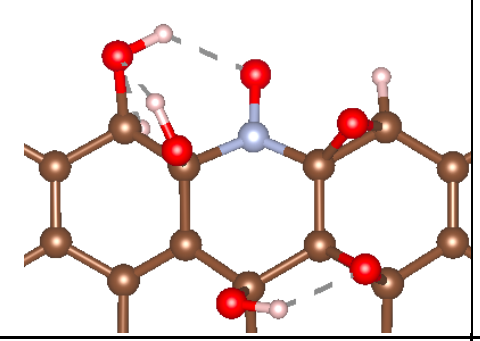	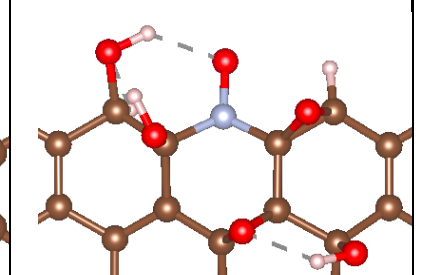
ΔE = 0.31	ΔE = 0.30	ΔE = 0.84	ΔE = 0.13	ΔE = 0.03	**ΔE = 0**

Table S8d: Relative energies of all OOH* configurations explored for the ZP-O/OH*+O*+OH* → ZP-O/OH*+O*+OH*+OOH* step. The most stable site has ΔG = 0.57 eV at 1.23 V-RHE and this value is higher in free energy than the preferred step in the current mechanism.

Site 1	Site 2	Site 3	Site 4	Site 5

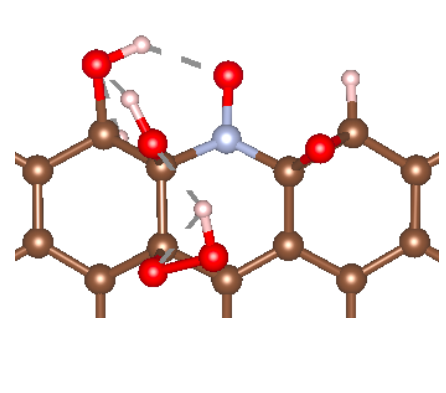	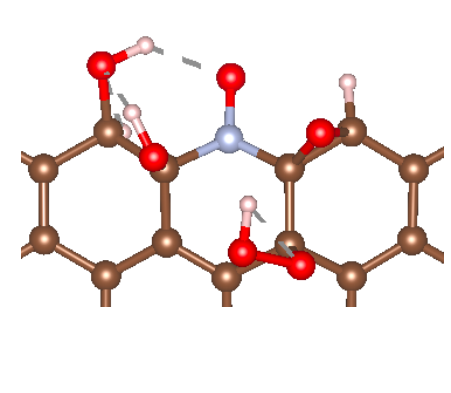	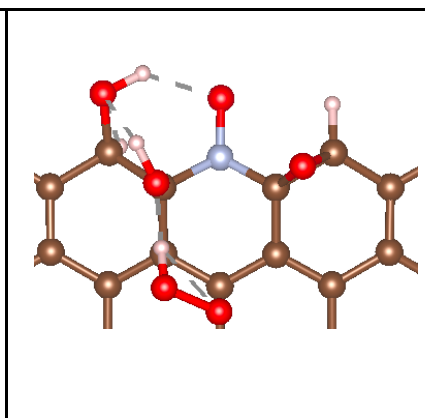	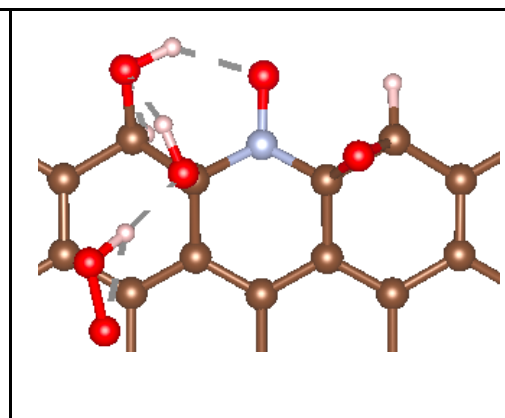	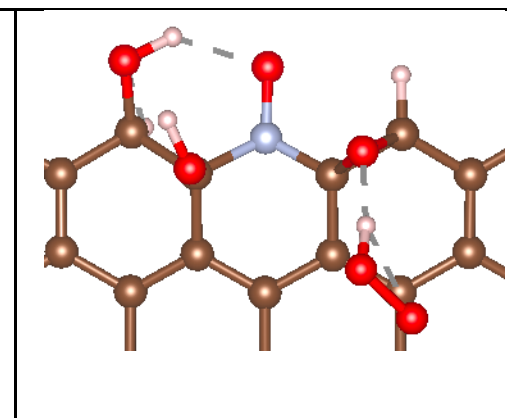
ΔE = 0.28	ΔE = 0.29	ΔE = 0.01	ΔE = 0.01	**ΔE = 0**

Since neither O*+OH* nor OOH* from the extended search is preferred over the existing preferred step, the mechanism proceeds via the existing pathway.

<u>Alternatives for the third step</u>

For third step, the preferred transition is another protonation of an existing OH* given by ZP-O/OH*+OH*+OH* → ZP-O/OH*+OH* + H_2O with a free energy change of 0.35 eV at 1.23 V-RHE. Below in Table S8e and S8f we examine if instead a O_2 can react to form an additional (O*+OH*) or (OOH*) respectively.

Table S8e: Relative energies of all O*+OH* configurations explored for the ZP-O/OH*+OH*+OH* → ZP-O/OH*+OH*+OH*+O*+OH* step. The most stable site has ΔG = 0.53 eV at 1.23 V-RHE and this higher in free energy than the preferred step in the current mechanism.

Site 1	Site 2	Site 3	Site 4	Site 5	Site 6
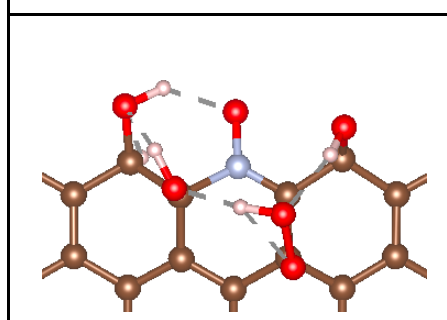	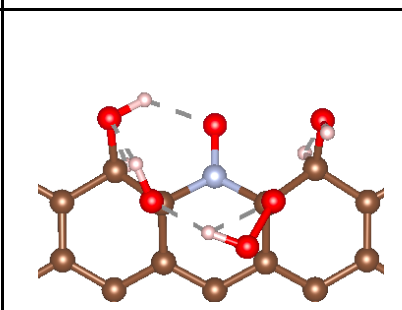	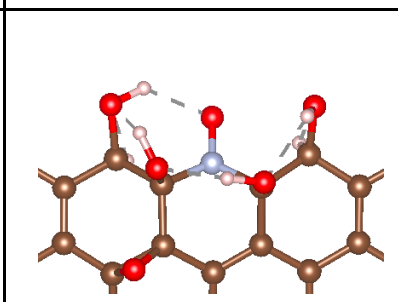	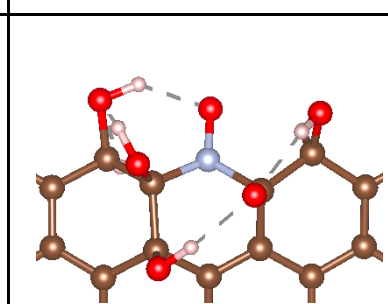	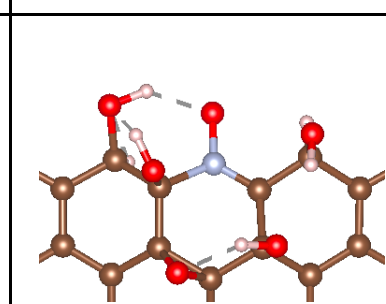	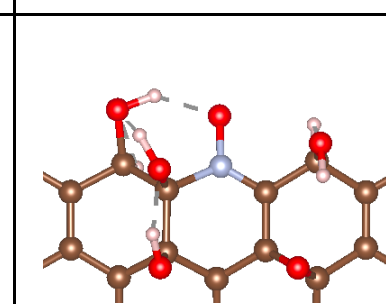
ΔE = 0.88	ΔE = 0.50	**ΔE = 0**	ΔE = 0.24	ΔE = 0.27	ΔE = 0.89

Table S8f: Relative energies of all OOH* configurations explored for the ZP-O/OH+OH*+OH* → ZP-O/OH*+OH*+OH*+OOH* step. The most stable site has ΔG = 1.17 eV at 1.23 V-RHE and this higher in free energy than the preferred step in the current mechanism.

Site 1	Site 2	Site 3	Site 4	Site 5
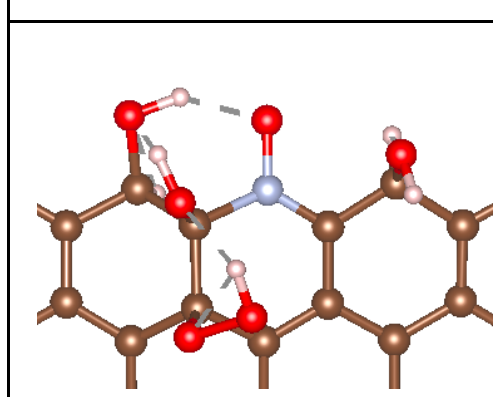	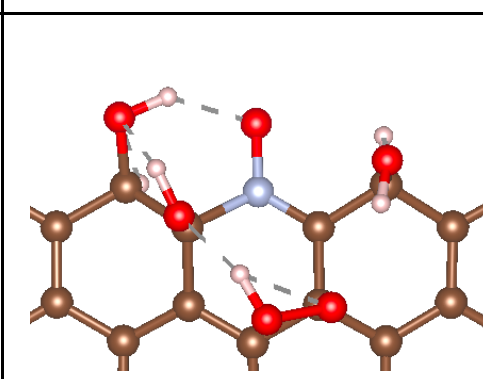	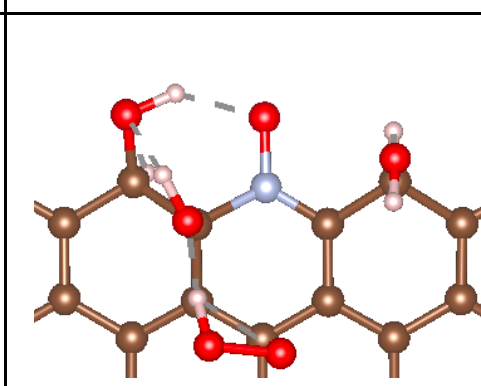	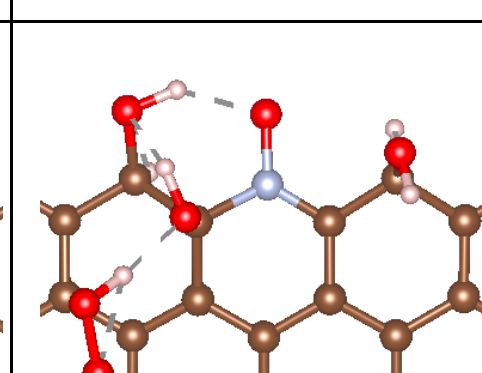	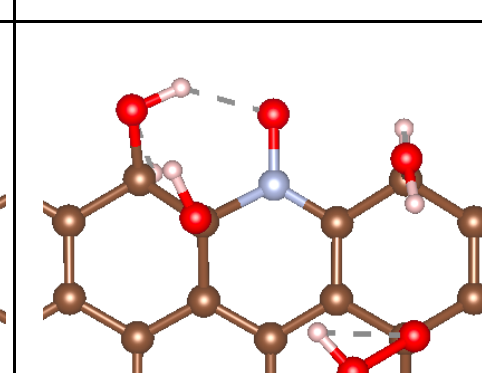
ΔE = 0.43	ΔE = 0.30	**ΔE = 0**	ΔE = 0.03	ΔE = 0.29

Again, since neither O*+OH* nor OOH* is preferred over the existing preferred step, the mechanism proceeds via the existing pathway.

<u>Alternatives for the fourth step</u>

Finally, the fourth and last step in the original cycle proceeds via another protonation of a existing OH* as given by ZP-O/OH*+OH* → ZP-O/OH* + H_2O with a free energy change of 0.14 eV at 1.23 V-RHE. Table S8g and S8h show alternative searches for O_2 to react to form (O*+OH*) and

OOH*, respectively. In a few of the attempts the (O*+OH*) recombines to OOH*, but overall, there are no new structures that are more favorable than the original preferred step.

Table S8g: Relative energies of all O*+OH* configurations explored for the ZP-O/OH*+OH* → ZP-O/OH*+OH*+O*+OH* step. The most stable site has ΔG = 0.53 eV at 1.23 V-RHE and this higher in free energy than the preferred step in the current mechanism.

Site 1	Site 2	Site 3	Site 4	Site 5
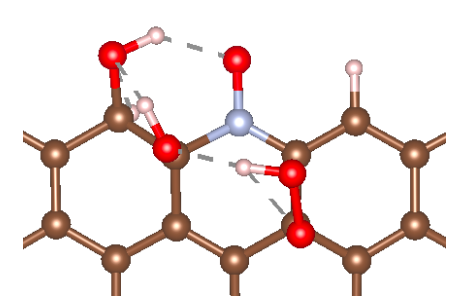	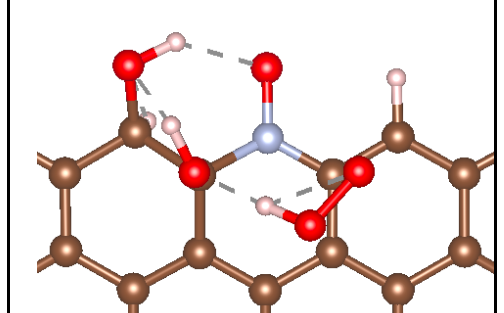	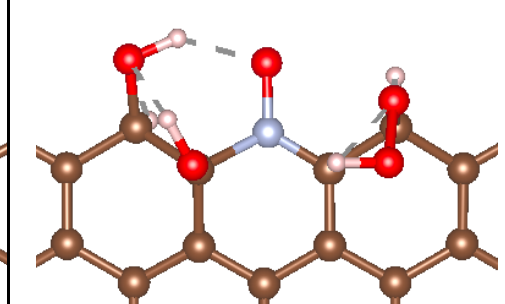	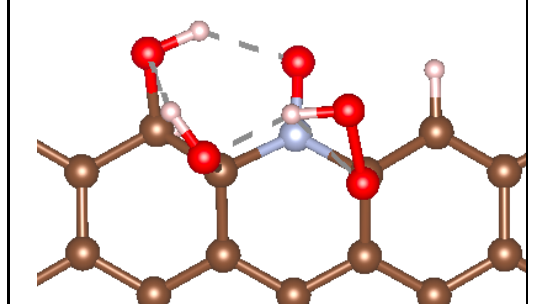	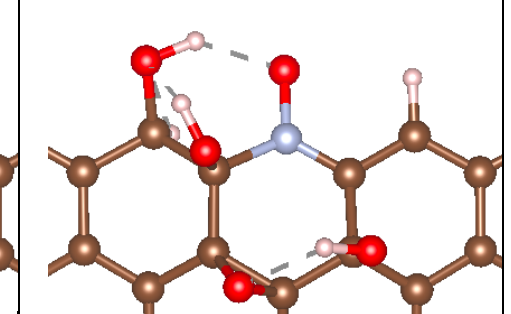
ΔE = 1.32	ΔE = 1.08	ΔE = 0.38	ΔE = 1.78	ΔE = 0.54
Site 6	Site 7	Site 8	Site 9	
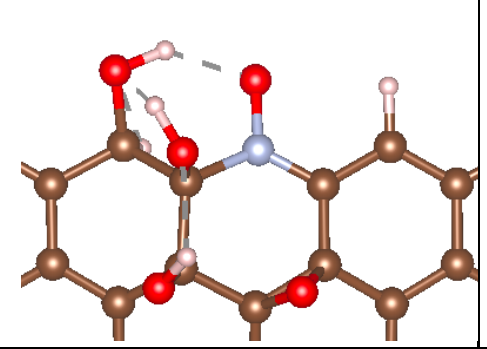	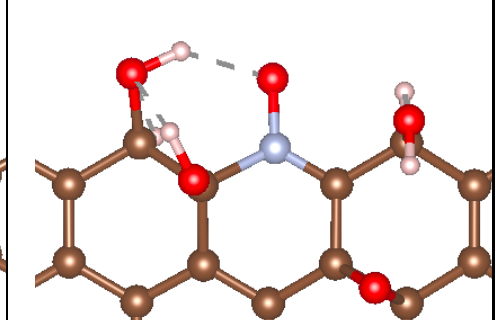	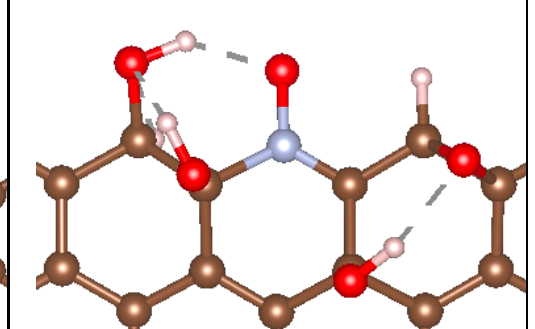	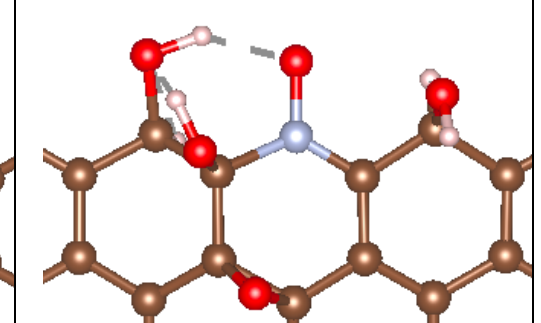	
ΔE = 0.71	**ΔE = 0**	ΔE = 0.28	ΔE = 0.17	

Table S8h: Relative energies of all OOH* configurations explored for the ZP-O/OH+OH* → ZP-O/OH*+OH*+OOH* step. The most stable site has ΔG = 0.78 eV at 1.23 V-RHE and is higher in free energy than the preferred step in the current mechanism.

Site 1	Site 2	Site 3	Site 4
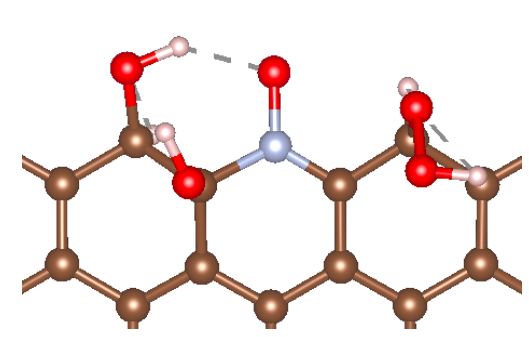	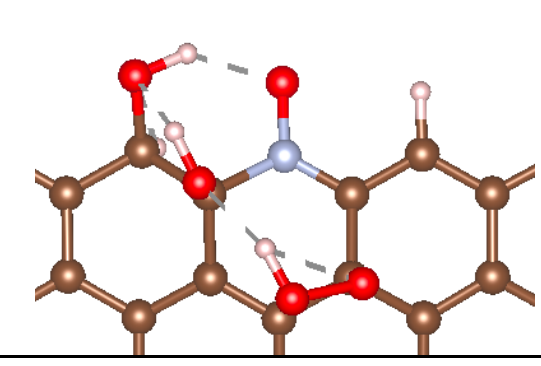	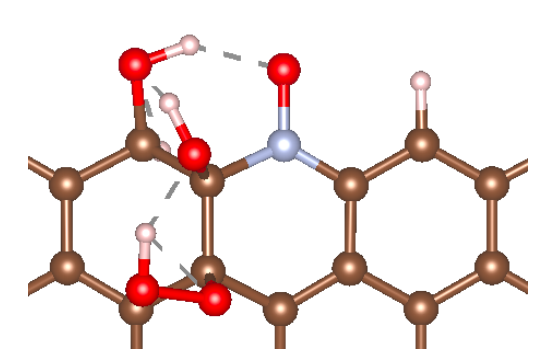	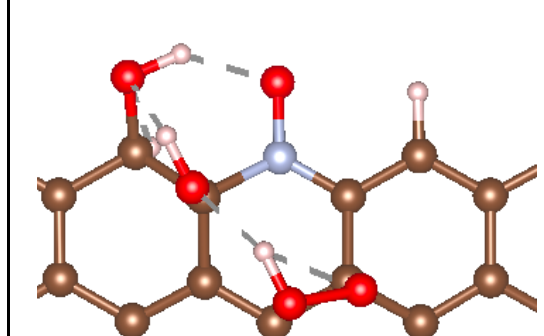
**ΔE = 0**	ΔE = 0.87	ΔE = 0.78	ΔE = 0.87

BQ additional search configurations

<u>Alternatives for the first step</u>

In the case of BQ, Figures 9c and Section S6 show that the preferred first step is BQ/O* → BQ/OH*, with a free energy change of 0.01 eV at 1.23 V-RHE. We examine additional (O*+OH*) or OOH* adsorption sites shown in Table S9a and S9b, respectively.

Table S9a: Relative energies of all O*+OH* configurations explored for the BQ/O* → BQ/O*+O*+OH* step. The most stable site has ΔG = 0.95 eV at 1.23 V-RHE and this structure was already identified in the existing integrated mechanism.

Site 1	Site 2	Site 3	Site 4	Site 5

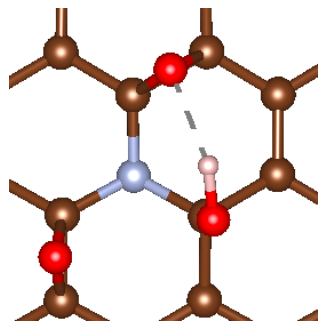	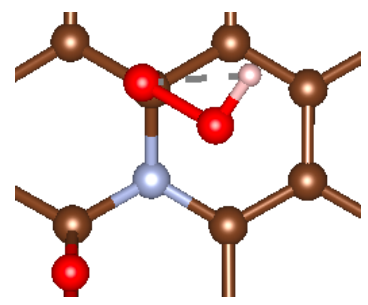	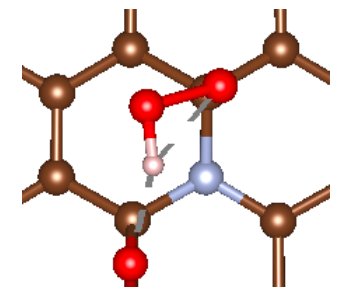	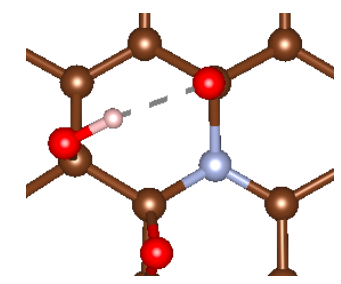	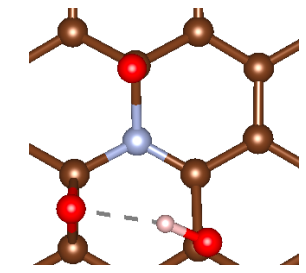
**ΔE = 0**	ΔE = 0.23	ΔE = 0.19	ΔE = 0.29	ΔE = 0.65

Table S9b: Relative energies of all OOH* configurations explored for the BQ/O* → BQ/O*+OOH* step. The most stable site has ΔG = 1.17 eV at 1.23 V-RHE and is higher in free energy than the preferred step in the current mechanism. Note for Site 1 and Site 3 that the H atom from OOH* is transferred to the surface O*. We consider this unphysical and do not include such configurations in the search, but generally they reflect the strong preference to protonate the surface O* versus other elementary steps.

Site 1	Site 2	Site 3	Site 4
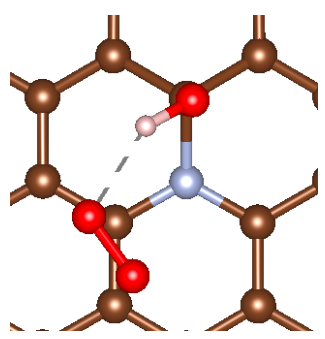	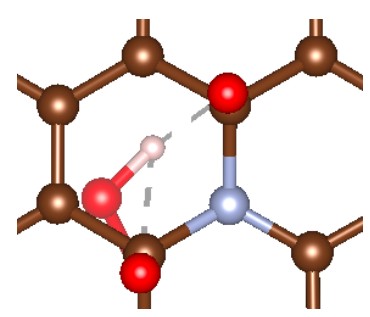	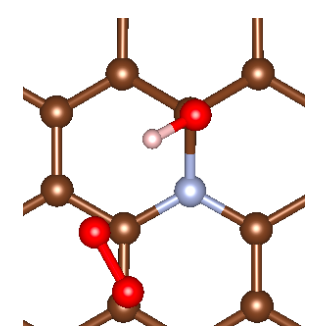	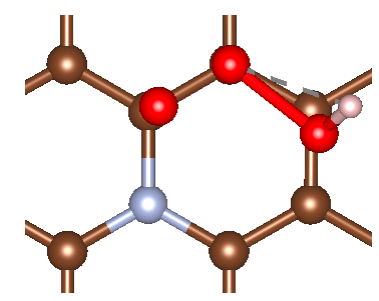
NA	**ΔE = 0**	NA	ΔE = 0.48

From exploration of additional adsorption sites, the conclusion for the first step remains unchanged, as all O*+OH* and OOH* configurations exhibit higher free energies than the preferred step.

<u>Alternatives for the second step</u>

The second preferred step is BQ/OH* → BQ + H_2O, with a free energy change of 0.18 eV at 1.23 V-RHE. We examine additional (O*+OH*) or OOH* adsorption sites shown in Table S9c and S9d, respectively.

Table S9c: Relative energies of all O+OH configurations explored for the BQ/OH* → BQ/OH*+O*+OH* step. The most stable site has ΔG = 0.18 eV at 1.23 V-RHE and matches the step in the current preferred mechanism.

Site 1	Site 2	Site 3	Site 4	Site 5
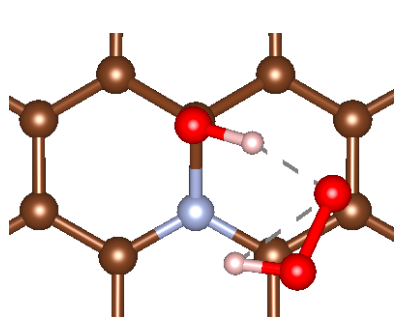	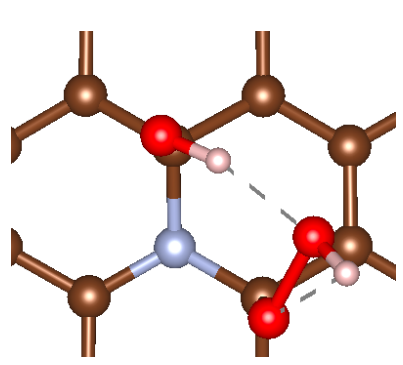	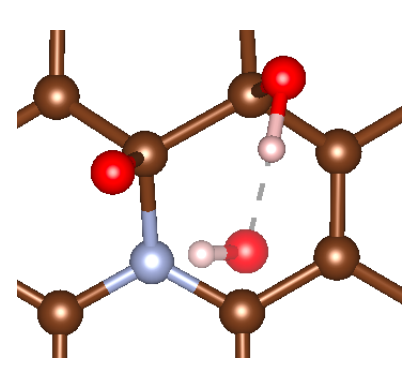	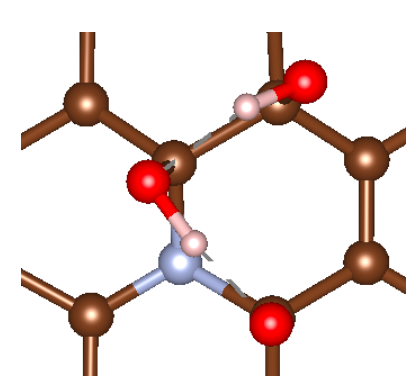	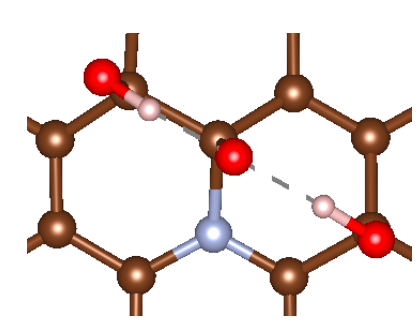
ΔE = 0.90	ΔE = 0.54	ΔE = 1.49	ΔE = 0.07	**ΔE = 0**

Table S9d: Relative energies of all OOH* configurations explored for the BQ/OH → BQ/OH*+OOH* step. The most stable site has ΔG = 0.52 eV at 1.23 V-RHE and is higher in free energy than the preferred step in the current mechanism.

Site 1	Site 2	Site 3	Site 4
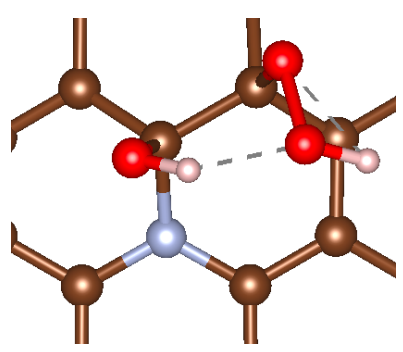	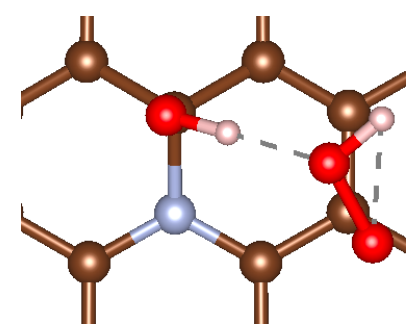	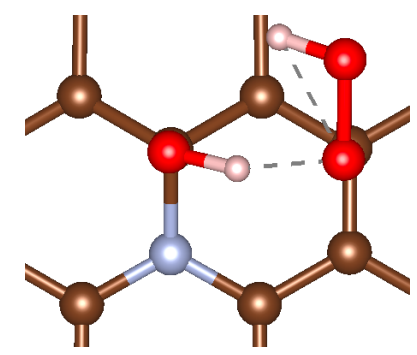	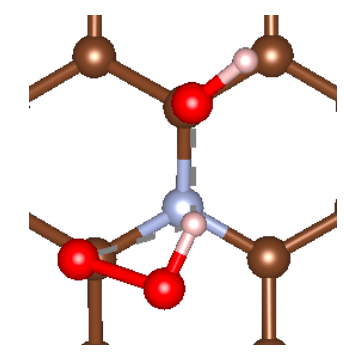
ΔE = 0.11	ΔE = 0.64	ΔE = 0.44	**ΔE = 0**

Since the free energy change of the explored O*+OH* and OOH* configurations is higher than that of the preferred step, the overall mechanistic picture remains unchanged, and we proceed to the next step in the cycle.

Alternatives for the third step

The third preferred step is BQ→ BQ/O*+OH*, with a free energy change of 0.24 eV at 1.23 V-RHE. We examine additional (O*+OH*) or OOH* adsorption sites that could compete with the identified step and these are shown in Table S9e and S9f, respectively.

Table S9e: Relative energies of all O*+OH* configurations explored for the BQ → BQ/O*+OH* step. The most stable site has ΔG = 0.24 eV at 1.23 V-RHE and matches the step in the current preferred mechanism.

Site 1	Site 2	Site 3	Site 4	Site 5	Site 6
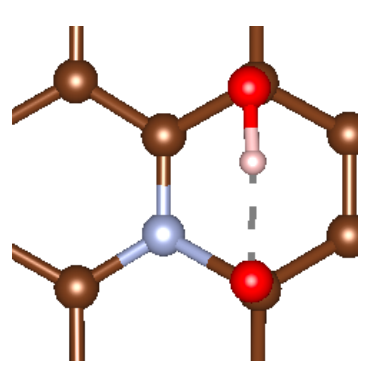	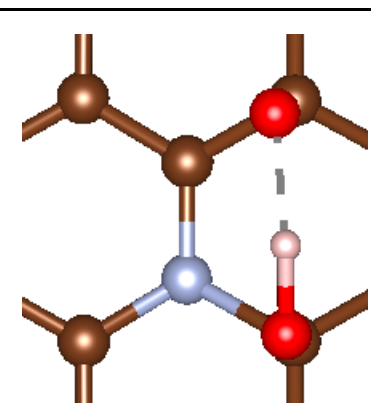	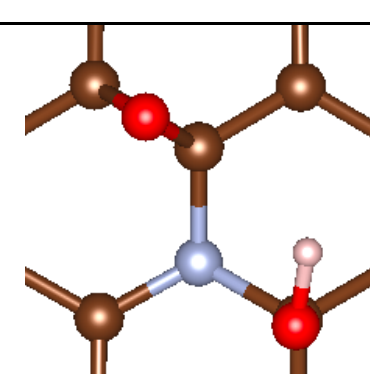	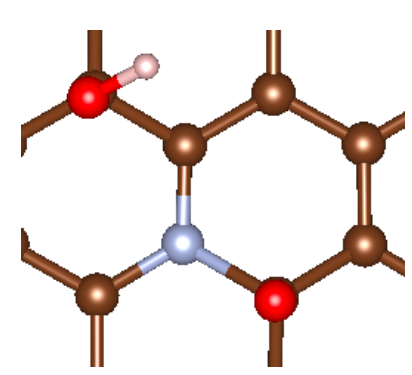	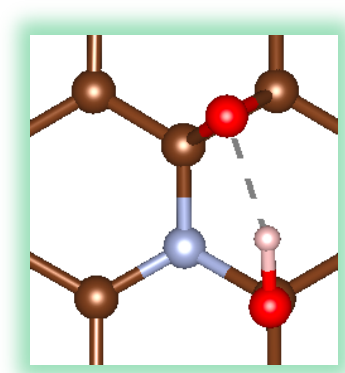	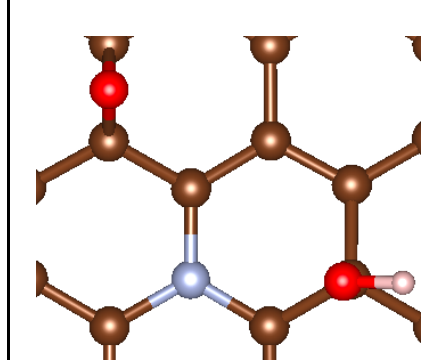
ΔE = 0.04	ΔE = 0.12	ΔE = 0.48	ΔE = 1.13	**ΔE = 0**	ΔE = 1.26

Table S9f: Relative energies of all OOH* configurations explored for the BQ → BQ/OOH* step. The most stable site has ΔG = 1.17 eV at 1.23 V-RHE and is higher in free energy than the preferred step in the current mechanism.

Site 1	Site 2	Site 3
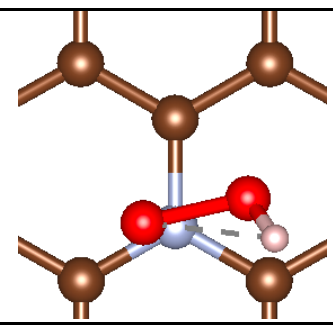	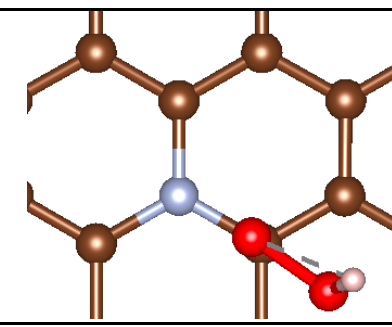	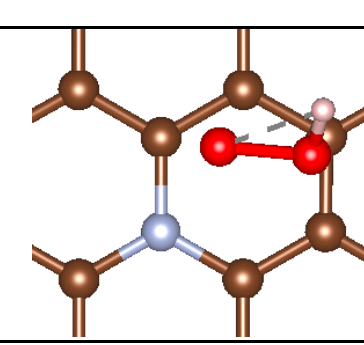
ΔE = 0.56	**ΔE = 0**	ΔE = 0.46

The extended site search for BQ identifies BQ/O*+OH* with both intermediates adsorbed on C1 sites as the most stable configuration, consistent with the site-selection rules used in the original integrated ORR mechanism.

<u>Alternatives for the fourth step</u>
The final (and fourth) preferred step is BQ/O*+OH* → BQ/O* + H_2O with a free energy change of -0.45 eV at 1.23 V-RHE. We examine additional (O*+OH*) or OOH* adsorption sites that could compete with the identified step and these are shown in Table S9g and S9h, respectively.

Table S9g: Relative energies of all O*+OH* configurations explored for the BQ/O*+OH* → BQ/O*+OH*+O*+OH* step. The most stable site has ΔG = 0.35 eV at 1.23 V-RHE and is higher in free energy than the preferred step in the current mechanism.

Site 1	Site 2	Site 3	Site 4	Site 5	Site 6
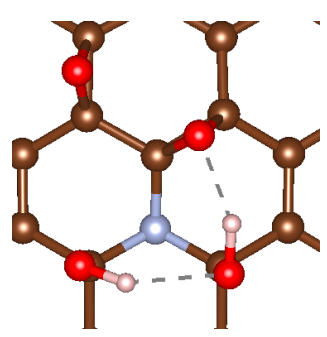	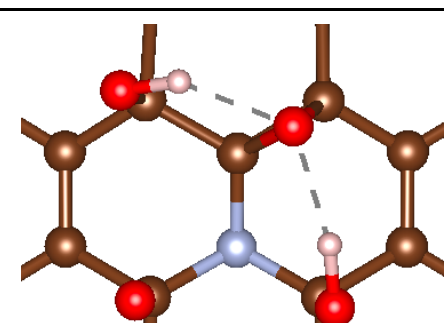	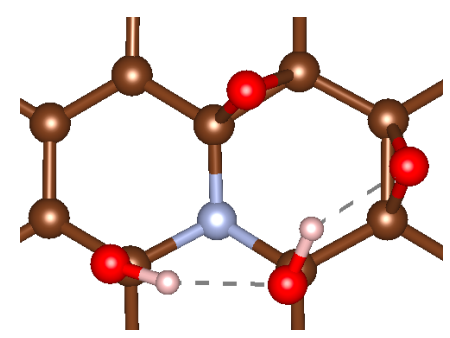	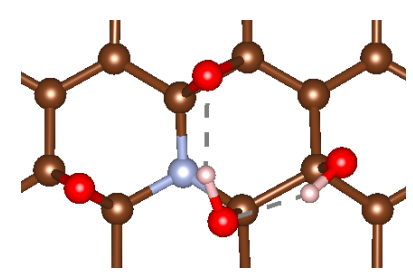	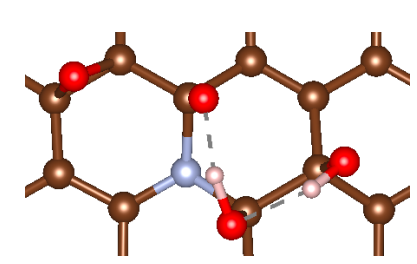	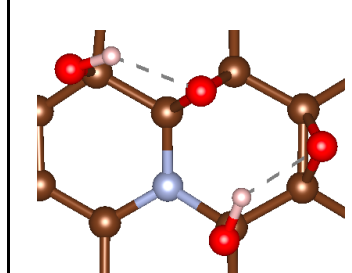
ΔE = 0.10	ΔE = 0.59	**ΔE = 0**	ΔE = 0.59	ΔE = 0.25	ΔE = 0.27

Table S9h: Relative energies of all OOH* configurations explored for the BQ/O*+OH* → BQ/O*+OH*+OOH* step. The most stable site has ΔG = 0.57 eV at 1.23 V-RHE and is higher in free energy than the preferred step in the current mechanism.

Site 1	Site 2	Site 3	Site 4
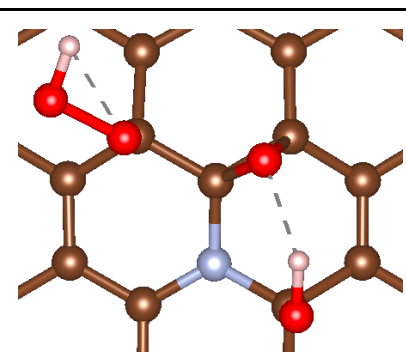	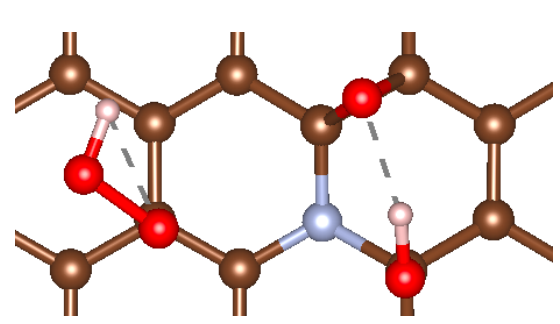	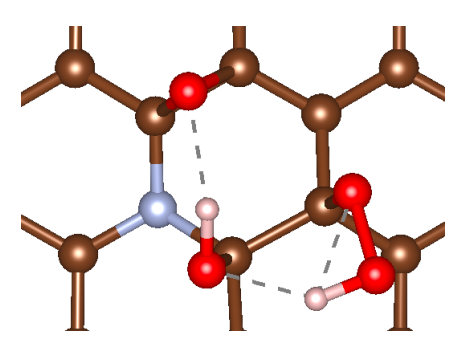	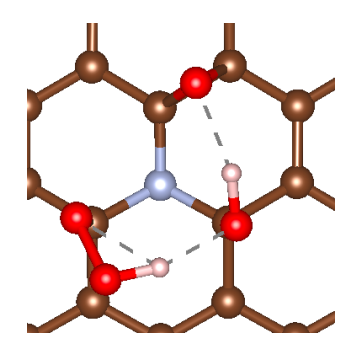
ΔE = 0.33	ΔE = 0.22	ΔE = 0.30	**ΔE = 0**

Once again, we find that exploration of additional adsorption sites does not alter the conclusions of the existing integrated ORR mechanism. From the extensive analysis carried out for both ZP and BQ, we conclude that the site-selection rule of considering OOH* and O*+OH* adsorption only on C1 sites is justified. Inclusion of adsorption sites beyond C1 does not identify more favorable configurations and therefore does not change the overall mechanistic picture.
